# Gut Microbial Composition of *Cyprinella lutrensis* (Red Shiner) and *Notropis stramineus* (Sand Shiner): Insights from Wild Fish Populations

**DOI:** 10.1007/s00248-024-02386-z

**Published:** 2024-05-22

**Authors:** Krista Starr, Federica Montesanto, Esther Perisho, Nirosh Aluthge, Mark Pegg, Samodha C. Fernando

**Affiliations:** University of NE–Lincoln, 3310 Holdrege Street, Lincoln, NE 68583 USA

**Keywords:** Gut microbiome, Bacterial community, Freshwater, Nebraska rivers, Metabolic pathways, Host-associated bacterial communities, Core microbiome, Small-bodied fish

## Abstract

The gut microbiome is a highly intricate ecosystem that exerts a pivotal influence on the host’s physiology. Characterizing fish microbiomes is critical to understanding fish physiology and health, but little is known about the ecology and colonization dynamics of microorganisms inhabiting fish species. In this study, we investigated the bacterial communities of two small-bodied fish species, *Cyprinella lutrensis* (red shiner) and *Notropis stramineus* (sand shiner), two fish species where gut microbiomes have not been investigated previously and surrounding waters, collected from rivers in Nebraska, USA. Our study focused on evaluating microbial diversity in small-bodied fish and identifying autochthonous microbes present within these species irrespective of location to better understand bacterial community composition and possible roles of such bacterial species. Our results revealed that both red shiner and sand shiner exhibited gut bacterial communities dominated by typical bacterial phyla found in freshwater fish. The phylum Bacteroidota was minimally abundant in both species and significantly lower in relative abundance compared to the surrounding water microbial community. Furthermore, we found that the gut microbiomes of red shiner and sand shiner differed from the microbial community in the surrounding water, suggesting that these fish species contain host-associated bacterial species that may provide benefits to the host such as nutrient digestion and colonization resistance of environmental pathogens. The fish gut bacterial communities were sensitive to environmental conditions such as turbidity, dissolved oxygen, temperature, and total nitrogen. Our findings also show bacterial community differences between fish species; although they shared notable similarities in bacterial taxa at phyla level composition, ASV level analysis of bacterial taxa displayed compositional differences. These findings contribute to a better understanding of the gut bacterial composition of wild, freshwater, small-bodied fish and highlight the influence of intrinsic (host) and environmental factors on shaping the bacterial composition.

**Supplementary Information:**

The online version contains supplementary material available at 10.1007/s00248-024-02386-z.

## Background

The gut microbiome is a rich and complex ecosystem that is tightly associated with its host, where the microbiome plays a significant role in modulating the host’s physiology and, in turn, the host provides nutrients and a hospitable environment to support survival of the gut microbes [1, 2]. The activity of gut microbes depends on their composition and diversity, which can affect the host’s feeding, digestion, and metabolism [1, 3, 4]. Furthermore, gut microbes also influence stress response, reproduction, development, and immune response [1, 3].

Extensive research efforts have been directed toward unraveling the intricate interplay between the genetic and environmental factors that shape the microbial communities residing in the gut [5]. Several studies on aquatic organisms have demonstrated that genetic divergence among hosts strongly shapes the taxonomic composition of the gut microbiome [6, 7], thus indicating that host genetics and physiology have a role in the assembly and persistence of gut microbiota communities. However, freshwater fish are in constant and direct contact with the aquatic environment. The complex and ever-changing microbiota within the aquatic environment can profoundly impact fish health and development [6]. In freshwater ecosystems, the environments and host dietary patterns exert significant influences on the gut microbiota composition and function, creating substantial intraspecific variation in the gut microbiota communities [8]. Thus, it is essential to consider host species, environmental conditions, and dietary habits to understand microbial communities associated with aquatic organisms.

The family Cyprinidae is the most diverse family of fishes with 285 genera and 3023 species globally [9]. Fish gut microbiome studies have primarily focused on fish species that may be reared in captivity because of the increasing demand for aquaculture products as well as for model fish species, such as *Oncorhynchus mykiss*, *Cyprinus carpio*, *Gadus morhua*, *Salmo salar*, *Ctenopharyngodon idella*, or the model species *Danio rerio* [1, 4, 10–12]. However, fewer studies have examined the gut microbiome of many small-bodied species collected from wild populations. Partly this is due to difficulty in sampling and collecting adequate gut contents for microbiome analysis. However, understanding how environmental conditions and diet affect microbiome establishment in wild small-bodied fish from different trophic levels may help understand how environmental and dietary factors help shape the microbiome in fish.

We investigated the gut microbiome of two cyprinid species, *Cyprinella lutrensis*, commonly known as red shiner, and *Notropis stramineus*, also known as sand shiner. *Cyprinella lutrensis* is a small-bodied freshwater species native to the Mississippi River basin from Wisconsin to Indiana and from South Dakota to Louisiana. Red shiners can grow to be 50–90 mm in size, while sand shiners tend to measure 40–82 mm. This species is characterized by an omnivorous feeding strategy, consuming small invertebrates, plant matter, algae, and occasionally fish larvae [13, 14]. *Cyprinella lutrensis* is notable for its extreme tolerance to harsh environmental conditions including low dissolved oxygen and high temperatures [15].

*Notropis stramineus* is a widely distributed cyprinid species native to the Great Plains of North America, encompassing a vast range from the headwaters of the Platte River in Wyoming to the Rio Grande River drainage in the south, the Tennessee River drainage in the east, and the lower Red River of the North drainage in Canada in the north [16]. This species exhibits a preference for habitats with moderate water velocity and a high proportion of sand substrate, making it most abundant in such lotic systems [17]. *Notropis stramineus* is considered a generalized insectivore that feeds on a diverse range of terrestrial and aquatic invertebrates, yet it may exhibit seasonal detritivorous feeding behavior [17]. Both species serve an important role as forage fish for larger predators from a food-web perspective. However, populations of *N. stramineus* are declining because of human pressures, such as habitat fragmentation and degradation [18, 19] while *C. lutrensis* is a widespread and tolerant species that has been recorded as non-indigenous in 11 basins in the US [20].

Here, we report the first investigation of gut bacterial communities for these two small-bodied fish species (red and sand shiner). This study was designed to describe microbial community composition in small-bodied fish species *N. stramineus* and *C. lutrensis* in Nebraska and to help assess host and environmental factors that lead to microbial community composition in small-bodied fish. Additionally, as these two species are phylogenetically closely related and are within the same trophic level, we were interested in intrinsic factors that may affect the structure of the microbial community. As such, in this study, we investigated the influence of intrinsic and extrinsic factors on gut bacterial composition and metabolic potential.

## Material and Methods

### Study Area and Sample Collection

**Fig. 1:**
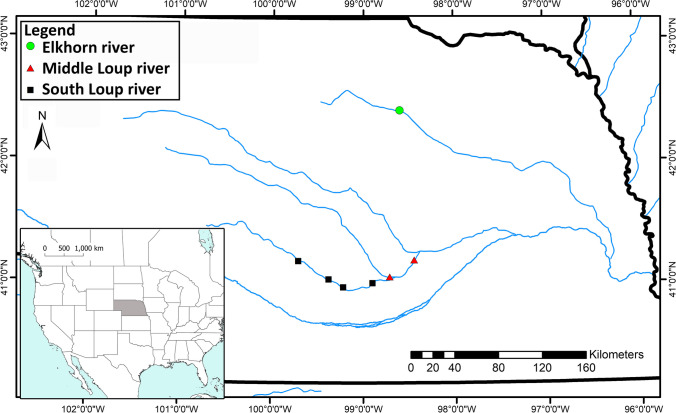
Map showing the sampling sites across Nebraska rivers (red rectangle highlighting the study area)

All animal procedures implemented in this study were approved by the University of Nebraska—Lincoln Animal Care and Use Committee under IACUC protocol 1746. All experiments were performed using relevant guidelines and regulations described by the Animal Care and Use Committee. Fish and water were collected at seven sites across two rivers in Nebraska, the Elkhorn and the Loup rivers (Fig. 1). One sampling site was located in the Elkhorn River, two along the Middle Loup River and four along the South Loup River. The Elkhorn River is located in north-eastern and north-central Nebraska and flows in the south-eastern direction until its confluence with the Platte River near Gretna, Nebraska, USA. Streamflow varies considerably along its entire length, which depends on snow melt, rainfall, and irrigation demands. The hydrogeology of the basin is complex due to the wide range of depositional environments from eolian in the west to glacial in the east, while the dominant land uses is cropland [21].

The Loup River is a tributary of the Platte River, approximately 109 km long; it flows eastward from its headwaters in the Sandhills of north-central Nebraska, through the central part of the state, before joining the Platte River near Columbus, Nebraska. The Loup River has two main tributaries that are commonly referred to as the South Loup River and the Middle Loup River. The streamflow of the Middle and South Loup Rivers is highly variable and is influenced by a variety of factors, including precipitation, snowmelt, and irrigation withdrawals, while the dominant land uses are cropland and pasture.

Water and fish samples were collected at the seven sites in June and July of 2019. For red shiner, a total of 26 samples were collected (Ravenna (6), Hwy183 (5), Presseywma (4), Boelus (3), O’Neil (3), South St. Paul (3), and Sartoria (2)). A total of 14 samples were collected for sand shiner (Presseywma (6), O’Neil (3), South St. Paul (3), Ravenna (1), and Sartoria (1)). A single water sample was collected from each sampling site at each sampling trip, with the exception of Presseywma, from which 2 samples were collected. Fish were captured by either a seine or with backpack and barge electrofishers. The fish were euthanized via an incision through the skull and were approximately > 50 mm in length for red shiners and > 40 mm in length for sand shiners. A sterile blade was used to slice down the abdomen of the fish, and the entire intestine was removed and used for microbial community analysis. The intestine sample was placed into a sterile 1.5 mL tube with sterile 0.2 mm zirconium silica beads and flash frozen in liquid nitrogen until arrival at the lab, at which point they were stored at − 80 ℃ until used for DNA extraction for bacterial community analysis. Water samples for bacterial community analysis were concurrently collected by filling a 500 mL autoclaved Nalgene bottle with water just under the surface of the river channel, away from the banks. The water samples were chilled on ice until they could be stored at − 20 ℃ in the lab. An additional 250 mL water sample was collected in a Nalgene bottle at each site to determine total nitrogen, total phosphorus, and nitrate levels of the river water. The water samples for nutrient assessment were stored on ice until shipment to Nebraska Water Center (1840 N 27th St, Lincoln, NE). Finally, water temperature, dissolved oxygen, turbidity, and ammonia were measured at each site. Water temperature and dissolved oxygen were recorded with a YSI handheld probe, while water clarity was measured with a turbidimeter. Digital pocket meters were used to measure ammonia.

### DNA Extraction and Sequencing of the 16S rRNA V4 Region

Water samples were centrifuged at 24,500 × *g* for 20 min to precipitate cells and DNA adapted from [22]. The supernatant was decanted, and the pellet was resuspended in 450 μL of 10 mM of Tris pH 8. DNA was extracted from the resuspended pellet using the Omega Mag-Bind® Stool DNA kit (Omega Bio-Tek) following the inhibitor rich protocol with the stated modifications. The lysis buffer was added directly to the resuspended pellet to start DNA extraction for the water samples. For the fish fecal samples, lysis buffer solution was added directly to the 1.5 mL tube containing fish intestine and sterile 0.2 mm zirconium silica beads. A TissueLyser (Qiagen Inc.) was used for 10 min at 20 Hz during cell lysis with sterile 0.2 mm zirconium silica beads to physically disrupt cell walls. Additionally, the cell lysate (between steps 14 and 15) was then subjected to DNA precipitation using 10 mM ammonium acetate and 100% isopropanol. The remainder of the Omega Mag-Bind inhibitor rich protocol was performed according to manufacturer instructions using a MagMax automated DNA extraction instrument (Thermo Fisher). The resulting DNA was evaluated for purity and quality using gel electrophoresis and Denovix spectrophotometer.

The 16S rRNA V4 region was amplified using polymerase chain reaction (PCR), and amplicons were prepared for sequencing on the Illumina MiSeq platform as described by Kozich et al. [23]. Briefly, the 25 μL PCR reactions consisted of 12.5 μL 2X Terra PCR Direct Buffer, 0.5 μL Terra PCR Direct Polymerase Mix, 400 nM of each barcoded primer, 2.0 μL DNA (20–50 ng of total DNA), and 9.0 μL sterile water. The thermal regime consisted of an initial denaturation at 98.0 ℃ for 3 min, followed by 30 cycles of 98 ℃ for 30 s, 55 ℃ for 30 s, and 68 ℃ for 45 s, with a final extension at 68 ℃ for 4 min. Following PCR, the amplicons were visualized using gel electrophoresis to check for correct fragment size and normalized for equal sample concentration using a NGS Normalization 96-Well Kit (Norgen Biotek Corp.). Normalized libraries were then pooled and sequenced using the Illumina MiSeq platform with 250 bp paired-end sequencing strategy using the V2 500 cycle kit.

Sequences were sorted into unique amplicon sequence variants (ASV) using the DADA2 [24] pipeline in R (version 4.1.1) [25]. Initial filtering of reads included trimming reads to keep only base pairs with a *Q*-score greater than 30. The maximum expected error rate was set to two. The representative ASVs generated were aligned to the SILVA reference database to assign taxonomic information to the ASVs identified [26] (version 138.1). Sequence abundances, taxonomy, and sample metadata were combined into a single dataset in R (version 4.2.2) using the package phyloseq (version 1.40.0) [27]. Sequences present in the negative controls resulting from reagent contamination were filtered out using the decontam package [28]. Amplicon sequence variants belonging to Eukaryota and Archaea were removed. Low abundance ASVs that may be PCR or sequencing artifacts were removed using the thresholds of minimum abundance of 0.15% and minimum prevalence of at least two samples. Rarefaction curves were generated to determine adequate read depth, and samples with less than 5000 reads were removed from the dataset.

### Statistical Analysis

Alpha diversity metric data distributions were assessed for normality graphically and by the Shapiro–Wilk normality test. If the alpha diversity metrics met the assumption of normality, groups were compared using parametric test statistics. If the metric failed to meet the normality assumption, non-parametric test statistics were employed. Significant results were considered values with *p* < 0.05 with the exception of the differential abundance test which used a significance cutoff of *p* < 0.01. An analysis of variance (ANOVA) model using Tukey’s honest significance test was used to test for variation in observed ASVs between sample types. Shannon index and Simpson indices were assessed using Kruskal–Wallis rank sum tests and post hoc Dunn tests [29]. A permutation analysis of variance (PERMANOVA) was performed to test for group clustering based on a Bray–Curtis dissimilarity matrix using the packages Vegan (version 2.6–4) and Microbiota Process (1.8.2) [30, 31]. As PERMANOVA is sensitive to unequal group variance with unbalanced study designs [32], we used BETADISPER to test for differences in group dispersion. Envfit in the Vegan package was used to test for linear relationships between environmental variables and the ASV diversity represented by the principal coordinate analysis (PCoA) axis [30]. Additionally, phylogenetically informed beta-diversity analysis was performed using weighted UniFrac analysis and non-metric multidimensional scaling (NMDS) plots.

Differential abundances between sample types were assessed at the phylum and family level using the DESeq2 package (version 1.36.0). DESeq2 utilized a negative binomial generalized linear model and the Wald test to identify taxa with differential abundance between groups. Differences were declared at *p* < 0.01 following adjustment using the Benjamini–Hochberg adjustment method [33]. Core microbiomes were identified as any ASV present in at least 80% of each sample type.

## Results

### Sample Collection

A total of 50 samples were collected including 40 fecal samples and 10 water samples (Table 1, and for sampling station, date, and further details, see Table S1 Supplementary Material). Initially, the sequencing results contained 913,606 reads. After post filtering and paired-end read assembly, a total of 789,624 reads were assessed belonging to 1205 ASVs. Post filtering samples contained an average of 15,792 reads (SD = 9756). Approximately, 0.5% of reads were assigned to archaea and 0.00033% of reads were assigned to eukaryotes and were removed from the dataset in subsequent analysis. Additionally, 26 potential contaminant ASVs (accounting for ~ 0.6% of total reads) were identified through “decontam” and were removed from the dataset from subsequent analysis.

**Table 1:** Feeding and habitat preference, collection river, and number of individuals collected from each river. Middle Loup River is abbreviated as Mloup and South Loup River is abbreviated as Sloup

				River		
Family	Species	Common name	Feeding-habitat*	Elkhorn	Mloup	Sloup
Cyprinidae	*Cyprinella lutrensis*	Red shiner	Insektivorous-pelagic	3	6	17
Cyprinidae	*Notropis stramineus*	Sand shiner	Insektivorous-benthic	3	3	8

*From FishBase database [13, 14, 34]

### Alpha and Beta-Diversity

Alpha diversity metrics of observed ASVs, Shannon diversity, and Simpson index were calculated for each sample. Water samples had the greatest observed number of ASVs (*M* = 258.10, SD = 40.98), Shannon index (*Mdn* = 4.67, SD = 0.10), and Simpson Index (*Mdn* = 0.98, SD = 0.00). The observed number of ASVs in each sample category was normally distributed, and the ANOVA model was significant (*F*_(2, 47)_ = 59.26). The Tukey test demonstrated that the water samples’ mean observed ASVs (258.10) are significantly higher than the mean observed ASVs for the fish samples. The mean observed ASVs in sand shiners were significantly higher than those in red shiners (*p* = 0.049). Shannon and Simpson indices had non-normal data distributions. The Kruskal–Wallis model for both Shannon index and Simpson index was significant. Shannon and Simpson index values indicated greater diversity in water than both red shiners (Shannon *p* = 0.00; Simpson *p* = 0.00) and sand shiners (Shannon *p* = 0.00; Simpson *p* = 0.00). Both the Shannon and Simpson index indicated no differences in alpha diversity between red shiners and sand shiners (*p* = 0.10, *p* = 0.21).

**Fig. 2:**
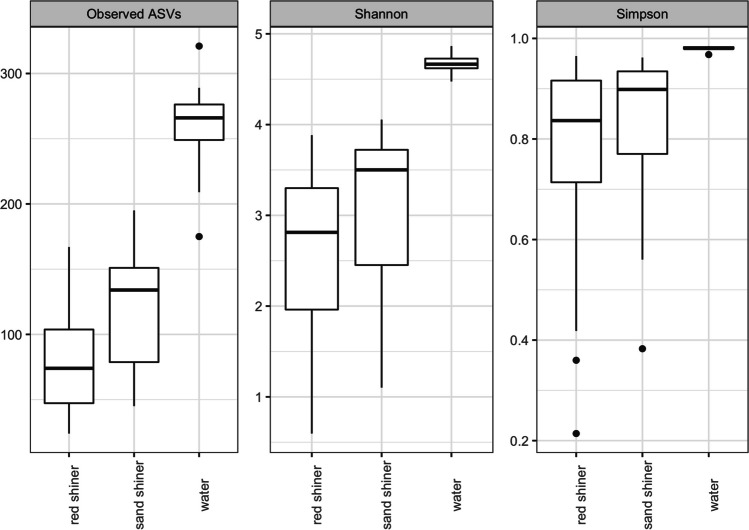
Alpha diversity of the bacterial community of sand shiner, red shiner, and the residing water column. Observed ASV numbers, Shannon diversity, and Simpson index for each sample type are shown as a box and whisker plot. The median is shown in the thick black bar with the edges of the box representing the upper and lower quartile. The upper and lower extremes are represented by the lines extending the bars and the dots represent outliers. Red shiner *n* = 26, sand shiner *n* = 14, water *n* = 10

There was no difference in the Shannon index between the two species (*p* = 0.101). Simpson index values were significantly higher in water than in fish samples. There was no difference in the Simpson index between the two species (*p* = 0.207) (Fig. 2).

Beta-diversity indicated significant clustering by sample type (*R*^2^ = 0.24) and river (*R*^2^ = 0.08). The interaction term of sample type by river was insignificant (*p* = 0.16). Pairwise PERMANOVA tests with Benjamini & Hochberg *p* value adjustments were performed between each sample type and river (Table S2). PERMANOVA test clearly indicated a weak but significant effect of the species identification on the bacterial community (*R*^2^ = 0.05, *p*-adj = 0.01). The BETADISPER model considering the sample type was significant (*F*_(2,47)_ = 26.46). Pairwise comparisons with Benjamini & Hochberg *p* value adjustments revealed that red shiners and sand shiner samples had more variance in bacterial community composition than water samples. There was no significant difference in dispersion between red shiner and sand shiner (*p* = 0.07). The BETADISPER model considering river was also significant (*F*_(2,47)_ = 5.76, *p* = 0.01) (Table S3). Post hoc testing revealed differences in variation between the South Loup and Elkhorn River samples as well as between the South Loup and Middle Loup River samples (Table S3).

### Core Microbiomes

**Fig. 3:**
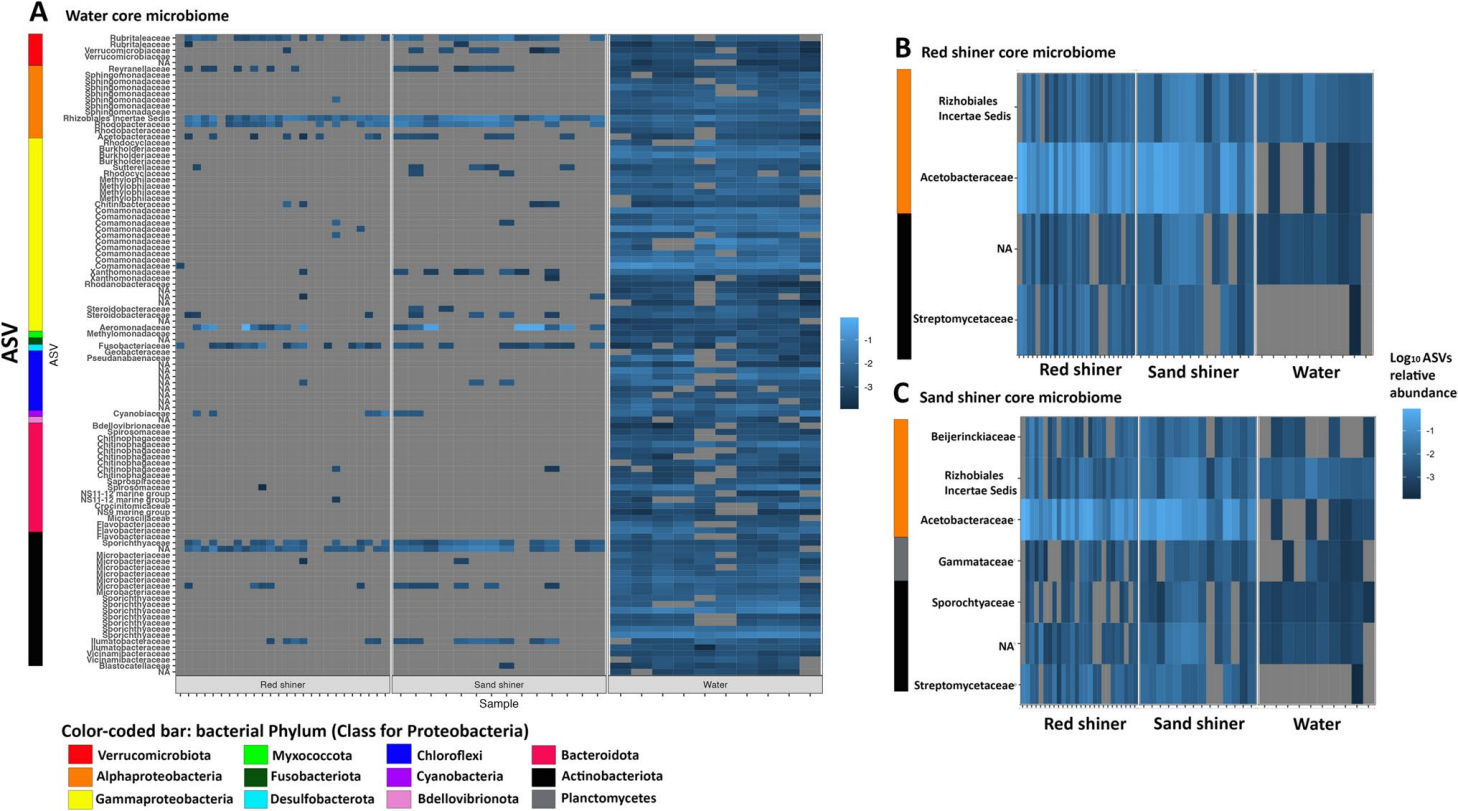
Core microbiome expressed as log_10_ (ASV relative abundance within a sample) belonging to **A** water samples (with comparisons with ASVs from the two fish species), **B** red shiner, and **C** sand shiner samples. NA = unknown family

Water samples contain a core microbiome of 104 ASVs (Table S4). The core microbiome of water samples accounts for 62% of the sequence reads in the water samples. *Actinobacteriota*, *Proteobacteria*, *Bacteroidota*, *Cyanobacteria*, and *Verrucomicrobiota* are the major phyla present in the water core microbiome. While some of the water-associated ASVs are common in fish samples, many are absent in the fish microbiomes or in very low abundance (Fig. 3A). Red shiners and sand shiners exhibit gut microbiomes dominated by typical bacterial phyla for freshwater fish including *Proteobacteria*, *Cyanobacteria*, *Firmicutes*, and *Actinobacteria* accounting large percentages of the fecal microbiome sequencing reads (min = 67.9%, max = 99.7%). Red shiner gut samples had a core microbiome including only four ASVs (Table S4). These four ASVs accounted for 27% of the sequence reads in red shiner samples. The phyla *Proteobacteria* and *Actinobacteriota* were present in the red shiner core microbiome (Fig. 3B). All four ASVs in the red shiner core microbiome were also found in the sand shiner core microbiome (Table S4). Sand shiner gut samples had a core microbiome of seven ASVs (Table S4). Those ASVs accounted for 24.04% of the sequence reads in sand shiner samples. The phyla *Proteobacteria*, *Actinobacteriota*, and *Planctomycetota* were found in the sand shiner core microbiome (Fig. 3C).

**Fig. 4:**
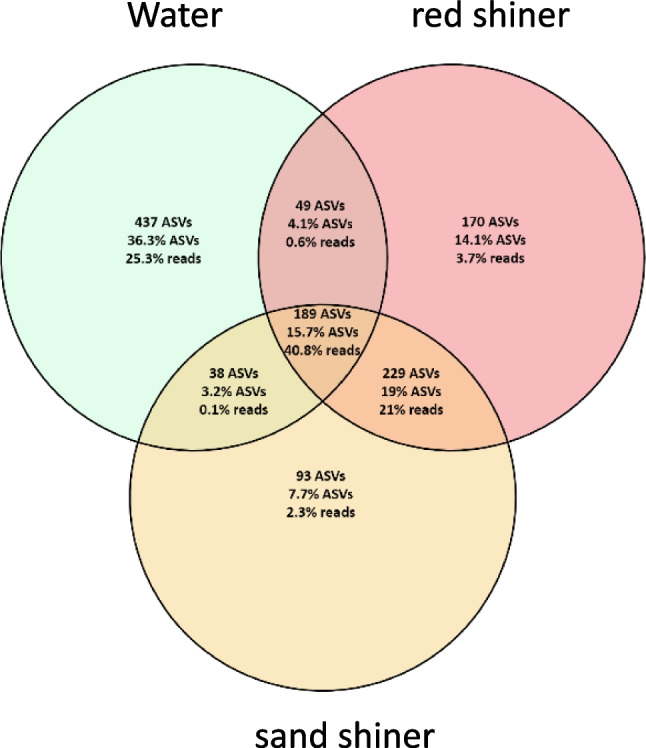
Venn diagram considering shared and unique taxa. Each section describes the number of ASVs, the percentage of ASVs in the dataset, and the percentage of sequence reads unique to the indicated sample types

The distribution of shared and unique taxa to each group was assessed by considering the number of ASVs, the percentage of ASVs, and the percentage of sequencing reads unique to specific sample types and shared between sample types. Water samples had a 437 ASVs and 25.3% of the sequencing reads which were not shared with either fish microbiome (Figs. 3 and 4). Among the ASVs found both in water samples and in fish microbiomes, 189 were in both red shiner and sand shiner (Figs. 3 and 4). ASVs that were found in water samples and only one of the fish species were rare with 49 ASVs found in red shiner and water samples only and 38 ASVs found in sand shiner and water samples only (Figs. 3 and 4). There were also 492 taxa found in the two shiner species but not in the water samples, 229 of which were found in both red shiner and sand shiners. However, each species maintained a subset of ASVs found uniquely in their respective microbiome (red shiner = 170 ASVs, sand shiner = 93 ASVs) (Figs. 3 and 4).

### Differential Abundance Results

**Fig. 5:**
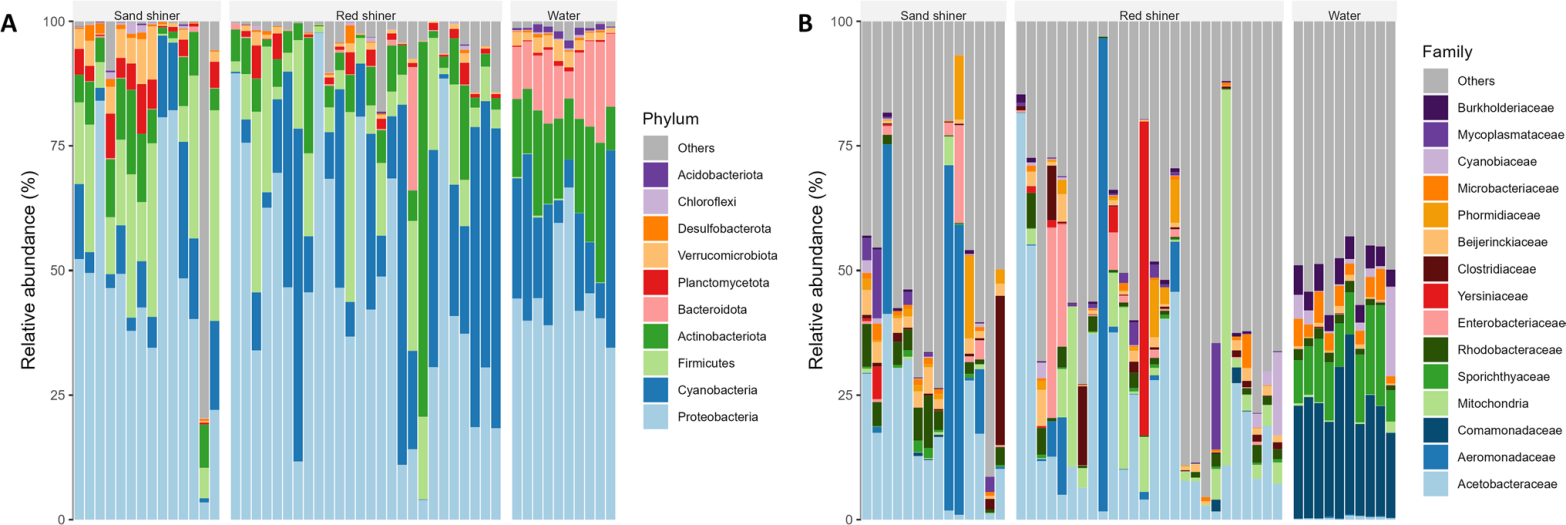
Relative abundance of phyla (**A**) and family (15 most abundant) (**B**) for fish (red shiner and sand shiners) and water samples. At the phylum level, any phylum that is less than 0.1% abundant in the dataset was considered “Other.” Differentially abundant ASVs between water column and each fish species and between fish species have been reported in supplementary material Tables S11–S13

We tested for taxa with differential abundance between water and fish bacterial communities at several taxonomic levels (phylum and family). Red shiner and sand shiners showed similar patterns of differential abundance at the phylum level. *Planctomycetota* and *Firmicutes* were more abundant in fish microbiomes than the surrounding water communities. Sand shiners additionally exhibited higher abundances of *Desulfobacterota* compared to the water samples. A variety of bacterial phyla were found in lower abundance in fish samples compared to the surrounding water showing potential negative selection, including *Gemmatimonadota*, *Armatimonadota*, *Bacteroidota*, *Acidobacteriota*, and *Bdellovibrionota* (Fig. 5; Table S5). Some phyla showed potential negative selection in only one fish species with red shiners having lower than expected *Actinobacteriota* and sand shiners having lower than expected *Latescibacterota*, *Myxococcota*, and *Myxococcota* (Fig. 4; Table S5). At the family level, red shiners had 71 differentially abundant taxa. *Streptomycetaceae* and *Eggerthellaceae* were more abundant in the red shiner bacterial community than the water, and the remaining 69 families were more abundant in the water samples. Sand shiners had 55 differentially abundant families with 11 families more abundant in the fish microbiome and 44 more abundant in the water community. Both fish species had higher abundances of *Streptomycetaceae* compared to the water samples. There were 44 bacterial families that were potentially selected against by both red shiners and sand shiners (Fig. 4; Table S6).

### Environmental Variables

**Fig. 6:**
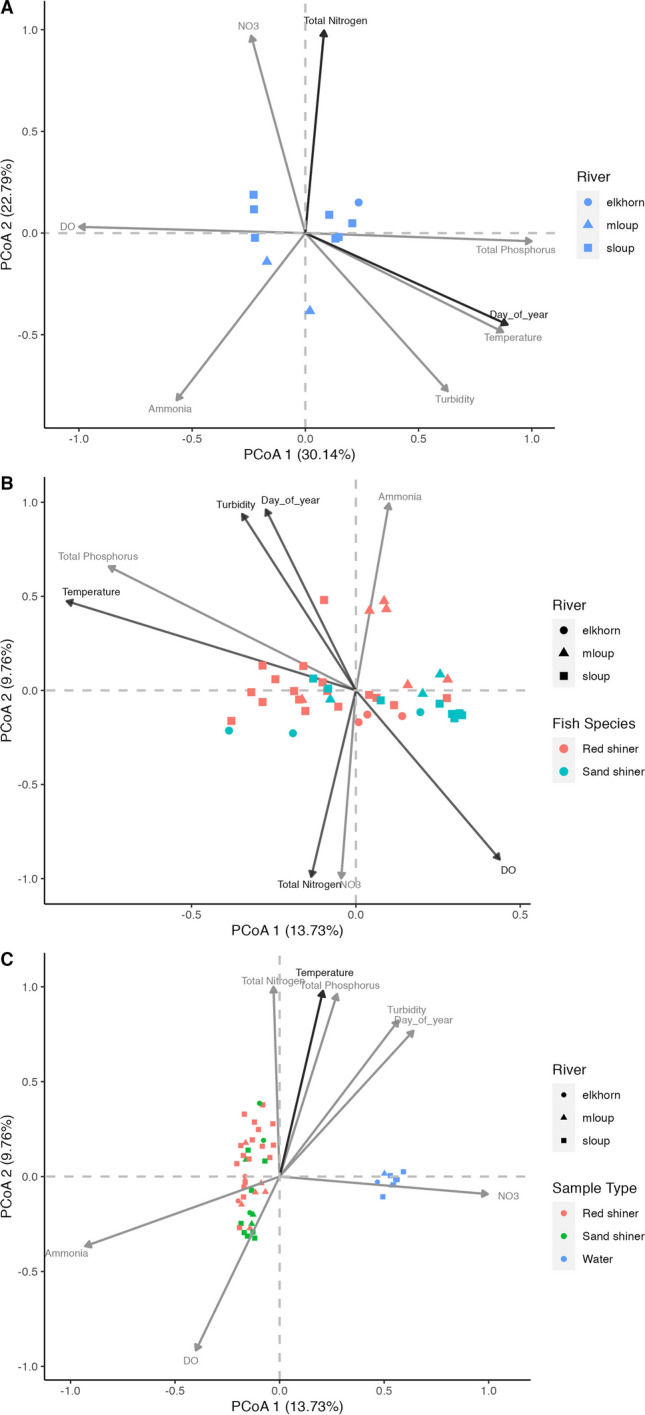
Principal component analysis for linear associations of environmental variables for **A** water samples, **B** fish (red shiners and sand shiners) samples, and **C** all samples collected across the 3 rivers. Significant environmental variables are shown in black and non-significant variables are shown in light gray. Middle Loup River is abbreviated as Mloup and South Loup River is abbreviated as Sloup

Water microbial community shifts were associated with changes in total nitrogen (*r*^2^ = 0.778, *p* = 0.007) and sampling date (*r*^2^ = 0.688, *p* = 0.021) (Fig. 6, Table S8). More environmental variables had significant impacts on fish microbiome communities including temperature, turbidity, dissolved oxygen, total nitrogen, and day of year (Fig. 6 and Supplementary Fig. 1, Table S9).

## Discussion

This study represents the first characterization of the gut microbiome of red shiners and sand shiners. Our results show that these two species exhibit gut microbiomes dominated by the bacterial phyla *Proteobacteria*, *Cyanobacteria*, *Firmicutes*, and *Actinobacteria*. Previous literature has shown mixed results on the dominance of the phylum *Bacteroidota* within freshwater fish gut microbiomes [4, 12, 35–37]. We found that the phylum *Bacteroidota* was minimally abundant in both fish microbiomes and was significantly lower in relative abundance than the surrounding water microbial community, indicating that gut conditions of these fish may exert selective pressure against microbes belonging to this phylum. The low prevalence of *Bacteroidota* is consistent with a previous study which found *Chrosomus neogaeus* microbiome to be dominated by *Firmicutes*, *Cyanobacteria*, *Planctomycetes*, and *Fusobacteria*. Our fish samples additionally exhibit similar tends of potential selection of gut microbiome taxa from the surrounding water column at the family level. Both species had higher abundances of a few bacterial taxa (red shiner *n* = 2, sand shiner *n* = 11) including the family *Streptomycetaceae* in both fish species. Forty-three bacterial families present in the water column were found at significantly lower relative abundance in both fish microbiomes.

Red shiners and sand shiners maintain a gut microbiome that is distinct from the microbial community in the surrounding water column. The samples exhibited clear differentiation between fish gut samples and water samples. Furthermore, many of the sequences found in fish fecal samples were not found in water samples taken at the same location and time (red shiner = 62.6%, sand shiner = 58.7%). Similar studies have also reported a majority of fish microbiome sequences being absent from water samples taken at the same time as the fecal samples [11, 38].

This likely indicates that fish selectively acquire microbes from the environment over time. Thus, studies interested in comparing the relative effects of host symbiosis vs environmental acquisition should consider investigating same host species under diverse environmental conditions or changing environmental conditions over time for the same fish population.

Moreover, fish gut microbiome samples exhibited more variation than the surrounding water microbial community and were apparent in the core microbiome comparison across water samples and fish gut samples (Fig. 3). All four ASVs in the red shiner core microbiome were also found in the sand shiner core microbiome suggesting that these 4 ASVs maybe host adapted bacterial strains of these fish species. Among the 4 bacterial ASVs identified, two were classified to the genre *Roseomonas* and *Streptomyces*. *Roseomonas* is a recently identified bacterial genera [39] that has been isolated from multiple environments [40–44]. Some species in these genera have been described as opportunistic pathogens of humans [45], but the role of this bacterial genera in fish species is unknown. The genus *Streptomyces* is known to produce bioactive compounds and has been suggested as a candidate for probiotics in aquaculture [46]. Studies have suggested siderophore producing *Streptomyces* sp. to influence colonization of pathogens such as *Vibrio* sp. [46]. Additionally, *Streptomyces* species produce inhibitory compounds and metabolites and can reduce pathogen colonization. As such, the presence of *Streptomyces* sp. as a core microbe in the fish gut may have health benefits to the host in reducing pathogen colonization.

There was a significant difference in the average distance from the group centroid between water samples and both fish species, indicating that fish gut microbiomes have higher variability than the surrounding water community (Fig. 6C and Supplementary Fig. 1C, Table S3). This difference in variability was observed in both the principal component analysis using the Bray–Curtis dissimilarity matrix (Fig. 6) and in the NMDS plots using weighted UniFrac distances (Supplementary Fig. 1). This is reflected in the number of ASVs considered as part of the core microbiome between the groups. The water samples had a core microbiome of 104 ASVs while red shiner samples identified only four core ASVs and sand shiners only seven. The influence of environmental variables differed between the fish fecal microbiomes and the water samples. Changes in the water microbial communities were associated with total nitrogen and sampling date throughout the year (samples collected June–July). However, changes in fish gut microbial communities were associated with gradients of turbidity, dissolved oxygen, temperature, total nitrogen, and sampling date throughout the year. Previous research indicates that fish gut microbiomes are influenced both by the environment and host selective factors resulting in differing microbial communities even between closely related species and species reared in the same environment [8, 47, 48]. Our results indicated many notable similarities between the gut microbiomes of the species analyzed. Both were dominated by the same four bacterial phyla, showed significantly more variability than the water microbial community, and selected against similar taxa present in the water microbial community. Nonetheless, our results indicated there are some, albeit subtle, differences between the gut microbiomes of the two species. For example, the phylum *Verrucomicrobiota* was more abundant in sand shiners, whereas the families *Ruminococcaceae*, *Eggerthellaceae*, and *Lactobacillaceae* were more abundant in red shiners. We hypothesize that the slight separation between red shiner and sand shiner gut microbiome communities is caused by the close phylogenetic relationship between the two species [49], as well as similar feeding diets in the shared habitats resulting in similar host associated selection factors.

## Conclusion

The present study highlights the importance of environmental and host factors in shaping gut bacterial communities in red shiners and sand shiners, two closely related cyprinid species, with overlapping preferred habitats and diet and widespread in central and western USA freshwater rivers, from Texas to southern Canada [50]. Further studies are needed to determine the effects of habitats and host phylogeny on gut bacterial composition in the examined species. Nevertheless, these results provide a first baseline information of gut bacterial composition of red shiners and sand shiners and also highlight potential host selection of gut bacterial communities.

### Supplementary Information

Below is the link to the electronic supplementary material.

Supplementary file1 (PNG 469 KB)

Supplementary file2 (DOCX 87 KB)

## Data Availability

The dataset supporting the conclusions of this article is available in the National Center for Biotechnology Information (NCBI) Sequence Read Archive (SRA) [BioProject accession no. PRJNA997952; https://www.ncbi.nlm.nih.gov/sra]. R code generated in the analysis of this data is available on GitHub [user: Samodha Fernando, repository: Fish-microbiome]. This study is reported in accordance with ARRIVE guidelines.

## Abbreviations

Mloup: Middle Loup River
Sloup: South Loup River
rRNA: Ribosomal ribonucleic acid
DNA: Deoxyribonucleic acid
PCR: Polymerase chain reaction
ASV: Amplicon sequence variant
ANOVA: Analysis of variance
PERMANOVA: Permutational analysis of variance
PCoA: Principal coordinate analysis
OTU: Operational taxonomic unit
KEGG: Kyoto encyclopedia of genes and genomes
