# Supplementary material for: Gut Microbial Composition of Cyprinella lutrensis (Red Shiner) and Notropis stramineus (Sand Shiner): Insights from Wild Fish Populations
Source: Microb Ecol. 2024 May 22;87:75. doi: 10.1007/s00248-024-02386-z (PMC11111511; doi:10.1007/s00248-024-02386-z)
Supplement: Supplementary file 1 [file 248_2024_2386_MOESM2_ESM.docx]

Table S1. River, station, coordinates and date of collection for fecal samples.

| River | Station | Latitude | Longitude | Date | Red shiner | Sand shiner |
| --- | --- | --- | --- | --- | --- | --- |
| elkhorn | oneil | 42.43304 | -98.599 | 7/2/2019 | 3 | 3 |
| mloup | soutstpaul | 41.20272 | -98.4461 | 6/24/2019 | 3 | 3 |
| mloup | boelus | 41.06476 | -98.7139 | 7/26/2019 | 3 | 0 |
| sloup | ravenna | 41.01308 | -98.9009 | 6/24/2019 | 5 | 0 |
| sloup | ravenna | 41.01308 | -98.9009 | 7/25/2019 | 1 | 1 |
| sloup | presseywma | 41.19295 | -99.7087 | 6/25/2019 | 1 | 5 |
| sloup | presseywma | 41.19295 | -99.7087 | 7/26/2019 | 3 | 1 |
| sloup | hwy183 | 41.04339 | -99.3789 | 6/25/2019 | 4 | 0 |
| sloup | hwy183 | 41.04339 | -99.3789 | 7/25/2019 | 1 | 0 |
| sloup | sartoria | 40.97865 | -99.22 | 7/25/2019 | 2 | 1 |

Table S2: PERMANOVA Analysis Results

| **Full model PERMANOVA** | | | | | | | | | | | |
| --- | --- | --- | --- | --- | --- | --- | --- | --- | --- | --- | --- |
|  | Df | Sum Sq | R^2^ | F | | | P | | | | |
| Sample type | 2 | 4.14 | 0.24 | 8.30 | | | 0.00 | | | | |
| River | 6 | 1.15 | 0.08 | 2.86 | | | 0.00 | | | | |
| Sample type : River | 4 | 1.15 | 0.07 | 1.16 | | | 0.16 | | | | |
| Residual | 41 | 10.23 | 0.60 |  | | | | |  |  |  |
| Total | 49 | 16.96 | 1.00 |  | | | | |  |  |  |
| **Sample type pairwise PERMANOVA** | | | | | | | | | | | |
|  | Red Shiner | Sand Shiner |  | | | |  |  |  |  |  |
| Sand Shiner | 0.01 |  | | |  |  |  |  |  |  |  |
| Water | 0.00 | 0.00 |  | | | |  |  |  |  |  |
| **River pairwise PERMANOVA** | | | | | | | | | | | |
|  | elkhorn | mloup |  | | | |  |  |  |  |  |
| mloup | 0.00 |  | | |  |  |  |  |  |  |  |
| sloup | 0.01 | 0.01 |  | | | |  |  |  |  |  |

Table S3. BETADISPER analysis results

| **Full models Betadisper** | | | | | |
| --- | --- | --- | --- | --- | --- |
|  | Df | Sum Sq | Mean Sq | F | P |
| Sample type | 2 | 0.38 | 0.19 | 26.46 | 0.000 |
| Residual | 47 | 0.34 | 0.01 |  |  |
| Station | 2 | 0.09 | 0.05 | 5.76 | 0.006 |
| Residual | 47 | 0.38 | 0.01 |  |  |
| **Sample type pairwise Betadisper** | | | | | |
|  | Red Shiner | Sand Shiner |  |  |  |
| Sand Shiner | 0.07 |  |  |  |  |
| Water | 0.00 | 0.00 |  |  |  |
| **River pairwise Betadisper** | | | | | |
|  | elkhorn | mloup |  |  |  |
| mloup | 0.61 |  |  |  |  |
| sloup | 0.01 | 0.02 |  |  |  |

Table S4. Core microbiomes of water, red and sand shiner samples.

| **ASV** | **Phylum** | **Class** | **Order** | **Family** | **Genus** |
| --- | --- | --- | --- | --- | --- |
| **Water** |  |  |  |  |  |
| ASV_107 | Actinobacteriota | Acidimicrobiia | Microtrichales | Ilumatobacteraceae | CL500-29 marine group |
| ASV_157 | Actinobacteriota | Acidimicrobiia | Microtrichales | Ilumatobacteraceae | CL500-29 marine group |
| ASV_5 | Actinobacteriota | Actinobacteria | Frankiales | Sporichthyaceae | NA |
| ASV_31 | Actinobacteriota | Actinobacteria | Frankiales | Sporichthyaceae | Candidatus Planktophila |
| ASV_152 | Actinobacteriota | Actinobacteria | Frankiales | Sporichthyaceae | Candidatus Planktophila |
| ASV_214 | Actinobacteriota | Actinobacteria | Frankiales | Sporichthyaceae | hgcI clade |
| ASV_12 | Actinobacteriota | Actinobacteria | Frankiales | Sporichthyaceae | hgcI clade |
| ASV_85 | Actinobacteriota | Actinobacteria | Frankiales | Sporichthyaceae | NA |
| ASV_40 | Actinobacteriota | Actinobacteria | Frankiales | Sporichthyaceae | hgcI clade |
| ASV_183 | Verrucomicrobiota | Verrucomicrobiae | NA | NA | NA |
| ASV_824 | Acidobacteriota | Acidobacteriae | Paludibaculum | NA | NA |
| ASV_387 | Proteobacteria | Gammaproteobacteria | Methylococcales | Methylomonadaceae | NA |
| ASV_4 | Proteobacteria | Gammaproteobacteria | Enterobacterales | Aeromonadaceae | Aeromonas |
| ASV_726 | Proteobacteria | Gammaproteobacteria | CCD24 | NA | NA |
| ASV_82 | Proteobacteria | Gammaproteobacteria | Steroidobacterales | Steroidobacteraceae | NA |
| ASV_122 | Proteobacteria | Gammaproteobacteria | Steroidobacterales | Steroidobacteraceae | NA |
| ASV_997 | Proteobacteria | Gammaproteobacteria | PLTA13 | NA | NA |
| ASV_184 | Proteobacteria | Gammaproteobacteria | PLTA13 | NA | NA |
| ASV_705 | Proteobacteria | Gammaproteobacteria | CCM19a | NA | NA |
| ASV_491 | Proteobacteria | Gammaproteobacteria | Xanthomonadales | Rhodanobacteraceae | Ahniella |
| ASV_215 | Proteobacteria | Gammaproteobacteria | Xanthomonadales | Xanthomonadaceae | Arenimonas |
| ASV_41 | Proteobacteria | Gammaproteobacteria | Xanthomonadales | Xanthomonadaceae | Arenimonas |
| ASV_2 | Proteobacteria | Gammaproteobacteria | Burkholderiales | Comamonadaceae | NA |
| ASV_9 | Proteobacteria | Gammaproteobacteria | Burkholderiales | Comamonadaceae | NA |
| ASV_42 | Proteobacteria | Gammaproteobacteria | Burkholderiales | Comamonadaceae | NA |
| ASV_94 | Proteobacteria | Gammaproteobacteria | Burkholderiales | Comamonadaceae | NA |
| ASV_44 | Proteobacteria | Gammaproteobacteria | Burkholderiales | Comamonadaceae | Ramlibacter |
| ASV_261 | Proteobacteria | Gammaproteobacteria | Burkholderiales | Comamonadaceae | NA |
| ASV_11 | Proteobacteria | Gammaproteobacteria | Burkholderiales | Comamonadaceae | Acidovorax |
| ASV_64 | Proteobacteria | Gammaproteobacteria | Burkholderiales | Comamonadaceae | Acidovorax |
| ASV_93 | Proteobacteria | Gammaproteobacteria | Burkholderiales | Comamonadaceae | Limnohabitans |
| ASV_20 | Proteobacteria | Gammaproteobacteria | Burkholderiales | Comamonadaceae | Limnohabitans |
| ASV_461 | Proteobacteria | Gammaproteobacteria | Burkholderiales | Chitinibacteraceae | Chitinibacter |
| ASV_327 | Proteobacteria | Gammaproteobacteria | Burkholderiales | Methylophilaceae | Methylotenera |
| ASV_71 | Proteobacteria | Gammaproteobacteria | Burkholderiales | Methylophilaceae | Methylotenera |
| ASV_188 | Proteobacteria | Gammaproteobacteria | Burkholderiales | Methylophilaceae | Methylotenera |
| ASV_58 | Proteobacteria | Gammaproteobacteria | Burkholderiales | Methylophilaceae | Candidatus Methylopumilus |
| ASV_288 | Proteobacteria | Gammaproteobacteria | Burkholderiales | Rhodocyclaceae | Dechloromonas |
| ASV_172 | Proteobacteria | Gammaproteobacteria | Burkholderiales | Sutterellaceae | NA |
| ASV_54 | Proteobacteria | Gammaproteobacteria | Burkholderiales | Burkholderiaceae | Polynucleobacter |
| ASV_3 | Proteobacteria | Gammaproteobacteria | Burkholderiales | Burkholderiaceae | Polynucleobacter |
| ASV_32 | Proteobacteria | Gammaproteobacteria | Burkholderiales | Burkholderiaceae | Polynucleobacter |
| ASV_102 | Proteobacteria | Gammaproteobacteria | Burkholderiales | Rhodocyclaceae | C39 |
| ASV_282 | Myxococcota | Polyangia | Blfdi19 | NA | NA |
| ASV_414 | Chloroflexi | KD4-96 | NA | NA | NA |
| ASV_504 | Proteobacteria | Alphaproteobacteria | Acetobacterales | Acetobacteraceae | Roseomonas |
| ASV_178 | Proteobacteria | Alphaproteobacteria | Rhodobacterales | Rhodobacteraceae | NA |
| ASV_39 | Proteobacteria | Alphaproteobacteria | Rhodobacterales | Rhodobacteraceae | NA |
| ASV_14 | Proteobacteria | Alphaproteobacteria | Rhizobiales | Rhizobiales Incertae Sedis | NA |
| ASV_377 | Proteobacteria | Alphaproteobacteria | Sphingomonadales | Sphingomonadaceae | Altererythrobacter |
| ASV_73 | Proteobacteria | Alphaproteobacteria | Sphingomonadales | Sphingomonadaceae | Novosphingobium |
| ASV_88 | Proteobacteria | Alphaproteobacteria | Sphingomonadales | Sphingomonadaceae | Novosphingobium |
| ASV_263 | Proteobacteria | Alphaproteobacteria | Sphingomonadales | Sphingomonadaceae | Novosphingobium |
| ASV_53 | Proteobacteria | Alphaproteobacteria | Sphingomonadales | Sphingomonadaceae | Sphingorhabdus |
| ASV_173 | Proteobacteria | Alphaproteobacteria | Sphingomonadales | Sphingomonadaceae | Sphingorhabdus |
| ASV_156 | Proteobacteria | Alphaproteobacteria | Sphingomonadales | Sphingomonadaceae | Rhizorhapis |
| ASV_22 | Bacteroidota | Bacteroidia | Flavobacteriales | Flavobacteriaceae | Flavobacterium |
| ASV_65 | Bacteroidota | Bacteroidia | Flavobacteriales | Flavobacteriaceae | Flavobacterium |
| ASV_47 | Bacteroidota | Bacteroidia | Flavobacteriales | Flavobacteriaceae | Flavobacterium |
| ASV_29 | Cyanobacteria | Cyanobacteriia | Synechococcales | Cyanobiaceae | Cyanobium PCC-6307 |
| ASV_60 | Cyanobacteria | Cyanobacteriia | Chloroplast | NA | NA |
| ASV_220 | Cyanobacteria | Cyanobacteriia | Chloroplast | NA | NA |
| ASV_312 | Cyanobacteria | Cyanobacteriia | Chloroplast | NA | NA |
| ASV_442 | Cyanobacteria | Cyanobacteriia | Chloroplast | NA | NA |
| ASV_81 | Cyanobacteria | Cyanobacteriia | Chloroplast | NA | NA |
| ASV_70 | Cyanobacteria | Cyanobacteriia | Chloroplast | NA | NA |
| ASV_18 | Cyanobacteria | Cyanobacteriia | Chloroplast | NA | NA |
| ASV_127 | Cyanobacteria | Cyanobacteriia | Chloroplast | NA | NA |
| ASV_388 | Proteobacteria | Alphaproteobacteria | Reyranellales | Reyranellaceae | Reyranella |
| ASV_421 | Acidobacteriota | Blastocatellia | Blastocatellales | Blastocatellaceae | Blastocatella |
| ASV_176 | Actinobacteriota | Actinobacteria | Micrococcales | Microbacteriaceae | MWH-Ta3 |
| ASV_84 | Actinobacteriota | Actinobacteria | Micrococcales | Microbacteriaceae | Aurantimicrobium |
| ASV_103 | Actinobacteriota | Actinobacteria | Micrococcales | Microbacteriaceae | Rhodoluna |
| ASV_72 | Actinobacteriota | Actinobacteria | Micrococcales | Microbacteriaceae | NA |
| ASV_116 | Actinobacteriota | Actinobacteria | Micrococcales | Microbacteriaceae | Rhodoluna |
| ASV_125 | Actinobacteriota | Actinobacteria | Micrococcales | Microbacteriaceae | Candidatus Planktoluna |
| ASV_112 | Actinobacteriota | Actinobacteria | Micrococcales | Microbacteriaceae | Candidatus Aquiluna |
| ASV_66 | Actinobacteriota | Actinobacteria | PeM15 | NA | NA |
| ASV_142 | Actinobacteriota | Actinobacteria | Frankiales | Sporichthyaceae | Longivirga |
| ASV_113 | Cyanobacteria | Cyanobacteriia | Pseudanabaenales | Pseudanabaenaceae | Pseudanabaena PCC-7429 |
| ASV_158 | Verrucomicrobiota | Verrucomicrobiae | Verrucomicrobiales | Verrucomicrobiaceae | Prosthecobacter |
| ASV_343 | Verrucomicrobiota | Verrucomicrobiae | Verrucomicrobiales | Verrucomicrobiaceae | NA |
| ASV_531 | Verrucomicrobiota | Verrucomicrobiae | Verrucomicrobiales | Rubritaleaceae | Luteolibacter |
| ASV_59 | Verrucomicrobiota | Verrucomicrobiae | Verrucomicrobiales | Rubritaleaceae | Luteolibacter |
| ASV_436 | Acidobacteriota | Vicinamibacteria | Vicinamibacterales | Vicinamibacteraceae | NA |
| ASV_360 | Acidobacteriota | Vicinamibacteria | Vicinamibacterales | Vicinamibacteraceae | NA |
| ASV_598 | Bdellovibrionota | Bdellovibrionia | Bdellovibrionales | Bdellovibrionaceae | OM27 clade |
| ASV_419 | Desulfobacterota | Desulfuromonadia | Geobacterales | Geobacteraceae | NA |
| ASV_51 | Fusobacteriota | Fusobacteriia | Fusobacteriales | Fusobacteriaceae | Cetobacterium |
| ASV_378 | Bacteroidota | Bacteroidia | Cytophagales | Microscillaceae | OLB12 |
| ASV_344 | Bacteroidota | Bacteroidia | Flavobacteriales | NS9 marine group | NA |
| ASV_96 | Bacteroidota | Bacteroidia | Flavobacteriales | Crocinitomicaceae | Fluviicola |
| ASV_286 | Bacteroidota | Bacteroidia | Sphingobacteriales | NS11-12 marine group | NA |
| ASV_333 | Bacteroidota | Bacteroidia | Sphingobacteriales | NS11-12 marine group | NA |
| ASV_19 | Bacteroidota | Bacteroidia | Cytophagales | Spirosomaceae | Pseudarcicella |
| ASV_74 | Bacteroidota | Bacteroidia | Chitinophagales | Saprospiraceae | Candidatus Aquirestis |
| ASV_242 | Bacteroidota | Bacteroidia | Chitinophagales | Chitinophagaceae | Dinghuibacter |
| ASV_316 | Bacteroidota | Bacteroidia | Chitinophagales | Chitinophagaceae | Dinghuibacter |
| ASV_193 | Bacteroidota | Bacteroidia | Chitinophagales | Chitinophagaceae | Dinghuibacter |
| ASV_331 | Bacteroidota | Bacteroidia | Chitinophagales | Chitinophagaceae | Lacibacter |
| ASV_218 | Bacteroidota | Bacteroidia | Chitinophagales | Chitinophagaceae | Sediminibacterium |
| ASV_46 | Bacteroidota | Bacteroidia | Chitinophagales | Chitinophagaceae | Sediminibacterium |
| ASV_154 | Bacteroidota | Bacteroidia | Chitinophagales | Chitinophagaceae | Sediminibacterium |
| ASV_253 | Bacteroidota | Bacteroidia | Cytophagales | Spirosomaceae | Lacihabitans |
| **Red Shiner** |  |  |  |  |  |
| ASV_1 | Proteobacteria | Alphaproteobacteria | Acetobacterales | Acetobacteraceae | Roseomonas |
| ASV_14 | Proteobacteria | Alphaproteobacteria | Rhizobiales | Rhizobiales Incertae Sedis | NA |
| ASV_36 | Actinobacteriota | Actinobacteria | Streptomycetales | Streptomycetaceae | Streptomyces |
| ASV_66 | Actinobacteriota | Actinobacteria | PeM15 | NA | NA |
| **Sand shiner** |  |  |  |  |  |
| ASV_1 | Proteobacteria | Alphaproteobacteria | Acetobacterales | Acetobacteraceae | Roseomonas |
| ASV_14 | Proteobacteria | Alphaproteobacteria | Rhizobiales | Rhizobiales Incertae Sedis | NA |
| ASV_61 | Proteobacteria | Alphaproteobacteria | Rhizobiales | Beijerinckiaceae | NA |
| ASV_36 | Actinobacteriota | Actinobacteria | Streptomycetales | Streptomycetaceae | Streptomyces |
| ASV_66 | Actinobacteriota | Actinobacteria | PeM15 | NA | NA |
| ASV_142 | Actinobacteriota | Actinobacteria | Frankiales | Sporichthyaceae | Longivirga |
| ASV_114 | Planctomycetota | Planctomycetes | Gemmatales | Gemmataceae | NA |

Table S5: DESEQ results table at the phylum level

| **Phylum** | **log2FoldChange** | **padj** |
| --- | --- | --- |
| **Red shiner vs water** |  |  |
| Gemmatimonadota | -7.02 | 0.000 |
| Armatimonadota | -5.52 | 0.000 |
| Bacteroidota | -5.37 | 0.000 |
| Acidobacteriota | -4.26 | 0.000 |
| Bdellovibrionota | -3.81 | 0.004 |
| Actinobacteriota | -0.96 | 0.006 |
| Planctomycetota | 2.34 | 0.001 |
| Firmicutes | 7.93 | 0.000 |
| **Sand shiner vs Water** |  |  |
| Gemmatimonadota | -8.08 | 0.000 |
| Armatimonadota | -6.52 | 0.000 |
| Bacteroidota | -5.7 | 0.000 |
| Latescibacterota | -5.6 | 0.001 |
| Bdellovibrionota | -4.12 | 0.003 |
| Myxococcota | -2.91 | 0.001 |
| Acidobacteriota | -2.69 | 0.001 |
| Desulfobacterota | 2.22 | 0.002 |
| Planctomycetota | 3.19 | 0.000 |
| Firmicutes | 7.38 | 0.000 |
| **Sand shiner vs Red shiner** |  |  |
| Verrucomicrobiota | 2.08 | 0.001 |

Table S6: DESEQ results table at the family level

| **Family** | **log2FoldChange** | **padj** |
| --- | --- | --- |
| **Red shiner vs Water** |  |  |
| Pseudohongiellaceae | -27.89 | 0.000 |
| Flavobacteriaceae | -16.40 | 0.000 |
| Methylophilaceae | -16.20 | 0.000 |
| Rhodocyclaceae | -15.50 | 0.000 |
| Crocinitomicaceae | -14.92 | 0.000 |
| Spirosomaceae | -14.68 | 0.000 |
| Pseudanabaenaceae | -14.52 | 0.000 |
| Gemmatimonadaceae | -13.39 | 0.000 |
| Vicinamibacteraceae | -13.38 | 0.000 |
| NS11-12 marine group | -13.25 | 0.000 |
| env.OPS 17 | -12.93 | 0.000 |
| Saprospiraceae | -12.88 | 0.000 |
| Armatimonadaceae | -12.65 | 0.000 |
| Hyphomonadaceae | -12.52 | 0.000 |
| Microscillaceae | -12.49 | 0.000 |
| Pedosphaeraceae | -12.36 | 0.000 |
| Chitinophagaceae | -12.13 | 0.000 |
| Alcaligenaceae | -11.85 | 0.000 |
| Nitrosomonadaceae | -11.59 | 0.000 |
| Cyclobacteriaceae | -11.52 | 0.000 |
| Blastocatellaceae | -11.47 | 0.000 |
| Rhodanobacteraceae | -11.40 | 0.000 |
| Steroidobacteraceae | -11.39 | 0.000 |
| Bdellovibrionaceae | -11.07 | 0.000 |
| Caulobacteraceae | -11.00 | 0.000 |
| Sutterellaceae | -10.91 | 0.000 |
| Geobacteraceae | -10.88 | 0.000 |
| Comamonadaceae | -10.83 | 0.000 |
| Bacteroidetes vadinHA17 | -10.83 | 0.000 |
| Sporichthyaceae | -10.82 | 0.000 |
| NS9 marine group | -10.67 | 0.000 |
| Desulfuromonadaceae | -10.59 | 0.000 |
| Burkholderiaceae | -10.50 | 0.000 |
| Devosiaceae | -10.16 | 0.000 |
| Ilumatobacteraceae | -10.10 | 0.000 |
| Unknown Family | -9.92 | 0.000 |
| Pyrinomonadaceae | -9.37 | 0.001 |
| Halieaceae | -9.32 | 0.000 |
| Xanthomonadaceae | -9.12 | 0.000 |
| Spongiibacteraceae | -9.04 | 0.007 |
| Cryomorphaceae | -8.95 | 0.001 |
| Verrucomicrobiaceae | -8.72 | 0.000 |
| Microbacteriaceae | -8.56 | 0.000 |
| Rickettsiaceae | -8.48 | 0.000 |
| Oxalobacteraceae | -8.33 | 0.000 |
| Reyranellaceae | -8.26 | 0.000 |
| Sphingomonadaceae | -7.79 | 0.000 |
| Chthoniobacteraceae | -7.62 | 0.000 |
| Chitinibacteraceae | -7.27 | 0.000 |
| Methylomonadaceae | -7.23 | 0.000 |
| Prevotellaceae | -6.96 | 0.002 |
| Gaiellaceae | -6.88 | 0.001 |
| Cyanobiaceae | -6.76 | 0.000 |
| Rubritaleaceae | -6.74 | 0.000 |
| Xanthobacteraceae | -6.63 | 0.000 |
| Iamiaceae | -6.35 | 0.000 |
| Methylococcaceae | -6.11 | 0.001 |
| Moraxellaceae | -5.96 | 0.000 |
| Rhizobiales Incertae Sedis | -5.76 | 0.000 |
| Hyphomicrobiaceae | -5.73 | 0.000 |
| Micrococcaceae | -5.69 | 0.001 |
| Isosphaeraceae | -5.45 | 0.004 |
| Rhodobacteraceae | -5.43 | 0.000 |
| Pseudomonadaceae | -5.36 | 0.001 |
| Rubinisphaeraceae | -5.00 | 0.000 |
| Beijerinckiaceae | -4.62 | 0.000 |
| Desulfocapsaceae | -3.99 | 0.002 |
| Rhizobiaceae | -3.94 | 0.010 |
| Gemmataceae | -3.07 | 0.000 |
| Streptomycetaceae | 2.85 | 0.000 |
| Eggerthellaceae | 19.47 | 0.000 |
| **Sand shiner vs Water** |  |  |
| Pseudohongiellaceae | -24.19 | 0.000 |
| Spirosomaceae | -12.64 | 0.000 |
| Pseudanabaenaceae | -11.55 | 0.000 |
| Flavobacteriaceae | -11.32 | 0.000 |
| Crocinitomicaceae | -11.26 | 0.000 |
| NS11-12 marine group | -10.95 | 0.000 |
| Comamonadaceae | -10.49 | 0.000 |
| Gemmatimonadaceae | -10.18 | 0.000 |
| Saprospiraceae | -10.10 | 0.000 |
| env.OPS 17 | -9.68 | 0.000 |
| Microscillaceae | -9.38 | 0.000 |
| Pedosphaeraceae | -8.89 | 0.000 |
| Hyphomonadaceae | -8.87 | 0.000 |
| Cyclobacteriaceae | -8.85 | 0.000 |
| Alcaligenaceae | -8.64 | 0.000 |
| Armatimonadaceae | -8.45 | 0.000 |
| Rhodanobacteraceae | -7.92 | 0.000 |
| Rhodocyclaceae | -7.86 | 0.000 |
| Chitinophagaceae | -7.80 | 0.000 |
| Unknown Family | -7.75 | 0.001 |
| Bacteroidetes vadinHA17 | -7.63 | 0.001 |
| Bdellovibrionaceae | -7.46 | 0.000 |
| Nitrosomonadaceae | -7.39 | 0.000 |
| Vicinamibacteraceae | -7.09 | 0.000 |
| Caulobacteraceae | -6.98 | 0.000 |
| Sporichthyaceae | -6.97 | 0.000 |
| NS9 marine group | -6.92 | 0.000 |
| Methylophilaceae | -6.10 | 0.002 |
| Blastocatellaceae | -6.03 | 0.000 |
| Cyanobiaceae | -5.71 | 0.000 |
| Rickettsiaceae | -5.71 | 0.003 |
| Burkholderiaceae | -5.71 | 0.000 |
| Chitinibacteraceae | -5.56 | 0.000 |
| Steroidobacteraceae | -5.32 | 0.000 |
| Sphingomonadaceae | -4.94 | 0.000 |
| Sutterellaceae | -4.89 | 0.000 |
| Xanthomonadaceae | -4.73 | 0.000 |
| Desulfuromonadaceae | -4.65 | 0.002 |
| Microbacteriaceae | -4.06 | 0.000 |
| Ilumatobacteraceae | -3.13 | 0.000 |
| Methylomonadaceae | -2.93 | 0.001 |
| Reyranellaceae | -2.28 | 0.000 |
| Verrucomicrobiaceae | -2.06 | 0.000 |
| Rhodobacteraceae | -1.08 | 0.001 |
| Pirellulaceae | 1.51 | 0.009 |
| Clostridiaceae | 3.23 | 0.001 |
| Bacillaceae | 3.48 | 0.002 |
| Acetobacteraceae | 3.72 | 0.000 |
| WX65 | 5.06 | 0.000 |
| Aeromonadaceae | 5.23 | 0.001 |
| Caldilineaceae | 5.61 | 0.005 |
| Streptomycetaceae | 6.22 | 0.000 |
| Erysipelotrichaceae | 7.95 | 0.000 |
| Enterococcaceae | 21.31 | 0.000 |
| Mycoplasmataceae | 24.26 | 0.000 |
| **Sand shiner vs Red shiner** |  |  |
| Ruminococcaceae | -24.99 | 0.000 |
| Eggerthellaceae | -23.67 | 0.000 |
| Lactobacillaceae | -9.10 | 0.001 |

Table S7: Environmental correlations with PCoA axis results including all sample types.

| **Variable** | **r^2^** | **p** |
| --- | --- | --- |
| ammonia | 0.003 | 0.942 |
| turbidity | 0.036 | 0.421 |
| disolved O_2_ | 0.053 | 0.271 |
| temperature | 0.151 | 0.018 |
| NO_3_ | 0.001 | 0.969 |
| total nitrogen | 0.005 | 0.887 |
| total phoshorous | 0.041 | 0.390 |
| day of year | 0.077 | 0.151 |

Table S8: Environmental correlations with PCoA axis results including only water samples.

| **Variable** | **r^2^** | **p** |
| --- | --- | --- |
| ammonia | 0.533 | 0.064 |
| turbidity | 0.087 | 0.760 |
| disolved O_2_ | 0.479 | 0.088 |
| temperature | 0.508 | 0.071 |
| NO_3_ | 0.429 | 0.139 |
| total nitrogen | 0.778 | 0.007 |
| total phoshorous | 0.025 | 0.893 |
| day of year | 0.688 | 0.021 |

Table S9: environmental correlations with PCoA axis results including only fecal samples.

| **Variable** | **r^2^** | **p** |
| --- | --- | --- |
| ammonia | 0.048 | 0.392 |
| turbidity | 0.155 | 0.042 |
| disolved O_2_ | 0.169 | 0.034 |
| temperature | 0.180 | 0.026 |
| NO_3_ | 0.079 | 0.217 |
| total nitrogen | 0.371 | 0.002 |
| total phoshorous | 0.072 | 0.254 |
| day of year | 0.324 | 0.003 |

Table S10: Water quality data for sampling events.

| **River** | **Station** | **Latitude** | **Longitude** | **Date** | **Ammonia** | **Turbidity** | **DO** | **Temperature** | **NO_3_** | **Total Nitrogen** | **Total Phosphorus** |
| --- | --- | --- | --- | --- | --- | --- | --- | --- | --- | --- | --- |
| elkhorn | oneil | 42.43304 | -98.59901 | 7/2/2019 | 0.2 | 28.23 | 5.43 | 26.4 | 0.508 | 1.762 | 0.275 |
| mloup | southstpaul | 41.20272 | -98.4461 | 6/24/2019 | 1.6 | 106.4 | 7.89 | 20.3 | 0.906 | 1.308 | 0.502 |
| mloup | boelus | 41.06476 | -98.71393 | 7/26/2019 | 0.71 | 71.37 | 5.54 | 24.8 | 0.26 | 0.525 | 0.203 |
| sloup | ravenna | 41.01308 | -98.90092 | 6/24/2019 | 1.14 | 71.89 | 7.36 | 23.7 | 1.35 | 1.744 | 0.419 |
| sloup | presseywma | 41.19295 | -99.70874 | 6/25/2019 | 0.16 | 68.62 | 7.45 | 17.9 | 1.836 | 2.12 | 0.299 |
| sloup | hwy183 | 41.04339 | -99.37889 | 6/25/2019 | 0.35 | 54.48 | 6.97 | 21 | 1.788 | 2.127 | 0.286 |
| sloup | ravenna | 41.01308 | -98.90092 | 7/25/2019 | 0.09 | 115.4 | 2.64 | 22.3 | 1.576 | 2.013 | 0.523 |
| sloup | sartoria | 40.97865 | -99.21999 | 7/25/2019 | 0.53 | 222 | 6.63 | 24 | 1.478 | 1.894 | 0.711 |
| sloup | hwy183 | 41.04339 | -99.37889 | 7/25/2019 | 0.52 | 87.44 | 6.5 | 26.6 | 1.676 | 2.065 | 0.346 |
| sloup | presseywma | 41.19295 | -99.70874 | 7/26/2019 | 0.4 | 59.39 | 6.57 | 21.2 | 1.957 | 2.457 | 0.295 |

Table S11: Differential ASVs identified between Sand shiner and Water samples

| **ASV_ID** | **log2FoldChange** | **padj** | **Lowest classification** |
| --- | --- | --- | --- |
| ASV_25 | -27.43125887 | 1.16E-20 | Cyanobacteriia |
| ASV_104 | 24.54572889 | 2.25E-20 | Bacilli |
| ASV_180 | -26.09942629 | 5.95E-20 | Comamonadaceae |
| ASV_342 | -25.72698891 | 1.84E-19 | Cyanobacteriia |
| ASV_95 | -25.36789769 | 2.05E-19 | *Sphaerotilus* |
| ASV_7 | -27.35177358 | 2.78E-18 | Cyanobacteriia |
| ASV_2 | -13.17053239 | 2.21E-17 | Comamonadaceae |
| ASV_43 | -26.44568332 | 3.37E-17 | Cyanobacteriia |
| ASV_5 | -12.61509092 | 1.24E-16 | Sporichthyaceae |
| ASV_407 | -25.5740622 | 1.24E-16 | Sporichthyaceae |
| ASV_141 | -25.31750882 | 1.24E-16 | *Cyanobium PCC-6307* |
| ASV_272 | 22.91054425 | 1.27E-16 | ZOR0006 |
| ASV_396 | -24.62799432 | 3.69E-16 | Comamonadaceae |
| ASV_638 | -24.69027945 | 3.69E-16 | *Polynucleobacter cosmopolitanus* |
| ASV_641 | -24.47710807 | 5.45E-16 | Microbacteriaceae |
| ASV_363 | -24.27757758 | 6.46E-16 | Hydrogenophaga |
| ASV_433 | -24.2468615 | 7.62E-16 | Algoriphagus |
| ASV_535 | -24.23308115 | 7.62E-16 | Sediminibacterium |
| ASV_349 | -24.05634686 | 9.15E-16 | Hydrogenophaga |
| ASV_700 | -24.11097865 | 1.03E-15 | Cyanobacteriia |
| ASV_1406 | -24.00215913 | 1.12E-15 | Pseudohongiellaceae |
| ASV_1297 | -24.0026271 | 1.12E-15 | Comamonadaceae |
| ASV_514 | -23.93128754 | 1.12E-15 | Oxalobacteraceae |
| ASV_149 | -23.933074 | 1.12E-15 | *Flavobacterium* |
| ASV_12 | -12.19979034 | 1.21E-15 | Sporichthyaceae |
| ASV_211 | 24.43392645 | 1.86E-15 | Cyanobacteriia |
| ASV_13 | -23.72206556 | 1.94E-15 | Cyanobacteriia |
| ASV_198 | 24.43255804 | 5.15E-15 | *Aeromonas salmonicida* |
| ASV_151 | 23.04536063 | 5.15E-15 | Candidatus Bacilloplasma |
| ASV_3 | -11.5820386 | 5.60E-15 | *Polynucleobacter asymbioticus* |
| ASV_299 | 24.21291761 | 8.82E-15 | *Aeromonas* |
| ASV_11 | -11.76372101 | 1.46E-14 | *Acidovorax facilis* |
| ASV_20 | -11.40183641 | 1.46E-14 | *Limnohabitans* |
| ASV_9 | -11.21906685 | 9.52E-14 | Comamonadaceae |
| ASV_17 | 23.13694441 | 1.39E-13 | Cyanobacteriia |
| ASV_37 | 22.82670945 | 3.09E-13 | Cyanobacteriia |
| ASV_19 | -11.51771876 | 3.93E-13 | *Pseudarcicella* |
| ASV_159 | 21.28517191 | 7.33E-13 | Cyanobacteriia |
| ASV_31 | -10.81673657 | 8.03E-13 | Candidatus Planktophila |
| ASV_18 | -11.15346931 | 1.03E-12 | Cyanobacteriia |
| ASV_32 | -10.73331035 | 2.23E-12 | *Polynucleobacter cosmopolitanus* |
| ASV_46 | -10.9221823 | 3.43E-12 | *Sediminibacterium* |
| ASV_65 | -10.5597257 | 9.89E-12 | *Flavobacterium cheonhonense* |
| ASV_58 | -10.18343534 | 2.15E-11 | Candidatus Methylopumilus |
| ASV_60 | -10.37435494 | 3.28E-11 | Cyanobacteriia |
| ASV_93 | -10.0306932 | 1.39E-10 | *Limnohabitans* |
| ASV_88 | -9.585854713 | 1.79E-10 | *Novosphingobium aromaticivorans* |
| ASV_71 | -9.769394502 | 2.00E-10 | *Methylotenera* |
| ASV_73 | -9.544015855 | 3.11E-10 | *Novosphingobium* |
| ASV_72 | -9.591427635 | 5.80E-10 | Microbacteriaceae |
| ASV_176 | -9.069707438 | 2.05E-09 | Microbacteriaceae |
| ASV_26 | 11.40189156 | 7.57E-09 | Bacilli |
| ASV_42 | -9.991865789 | 7.89E-09 | Comamonadaceae |
| ASV_113 | -10.66711856 | 1.61E-08 | Pseudanabaena PCC-7429 |
| ASV_103 | -9.788907037 | 1.87E-08 | *Rhodoluna* |
| ASV_44 | -11.08478729 | 3.10E-08 | *Ramlibacter* |
| ASV_40 | -9.555738272 | 6.20E-08 | Sporichthyaceae |
| ASV_282 | -8.530563768 | 6.33E-08 | Blfdi19 |
| ASV_70 | -10.70930582 | 9.53E-08 | Cyanobacteriia |
| ASV_53 | -9.286517003 | 1.00E-07 | *Sphingorhabdus* |
| ASV_54 | -9.24420404 | 1.53E-07 | *Polynucleobacter* |
| ASV_85 | -10.69898595 | 1.57E-07 | Sporichthyaceae |
| ASV_188 | -9.106273465 | 2.25E-07 | *Methylotenera* |
| ASV_116 | -9.125969899 | 2.35E-07 | *Rhodoluna* |
| ASV_107 | -8.243702117 | 2.46E-07 | *CL500-29 marine group* |
| ASV_158 | -7.828297905 | 2.76E-07 | *Prosthecobacter* |
| ASV_112 | -8.786836677 | 3.97E-07 | Candidatus Aquiluna |
| ASV_1 | 6.053898446 | 4.67E-07 | *Roseomonas* |
| ASV_94 | -10.06434603 | 4.77E-07 | Comamonadaceae |
| ASV_173 | -8.583857614 | 1.06E-06 | Sphingorhabdus |
| ASV_64 | -8.361842188 | 1.09E-06 | *Acidovorax carolinensis* |
| ASV_220 | -8.720045188 | 1.20E-06 | Cyanobacteriia |
| ASV_360 | -7.411987524 | 1.49E-06 | Vicinamibacteraceae |
| ASV_96 | -9.784727708 | 1.54E-06 | *Fluviicola* |
| ASV_387 | -7.662869902 | 1.64E-06 | Methylomonadaceae |
| ASV_178 | -8.305100621 | 2.06E-06 | Rhodobacteraceae |
| ASV_193 | -8.207947289 | 2.16E-06 | Dinghuibacter |
| ASV_47 | -9.329284526 | 2.70E-06 | Flavobacterium |
| ASV_377 | -8.124168271 | 3.65E-06 | Altererythrobacter |
| ASV_22 | -9.216589879 | 4.11E-06 | *Flavobacterium saccharophilum* |
| ASV_102 | -8.991298847 | 4.35E-06 | Rhodocyclaceae |
| ASV_156 | -8.238236852 | 5.36E-06 | Rhizorhapis |
| ASV_125 | -7.325494228 | 9.80E-06 | Candidatus Planktoluna |
| ASV_74 | -7.727717465 | 1.36E-05 | Candidatus Aquirestis |
| ASV_312 | -8.412502552 | 1.65E-05 | Cyanobacteriia |
| ASV_378 | -7.550170141 | 1.79E-05 | Microscillaceae |
| ASV_119 | -9.337873179 | 2.26E-05 | Comamonadaceae |
| ASV_263 | -8.230151258 | 2.26E-05 | *Novosphingobium* |
| ASV_239 | -9.654236823 | 2.26E-05 | Chitinophagaceae |
| ASV_218 | -8.327706741 | 2.38E-05 | *Sediminibacterium* |
| ASV_253 | -7.49086494 | 2.42E-05 | *Lacihabitans* |
| ASV_242 | -8.388863232 | 2.67E-05 | *Dinghuibacter* |
| ASV_152 | -8.151188836 | 3.19E-05 | Candidatus Planktophila |
| ASV_327 | -8.109826361 | 3.27E-05 | *Methylotenera* |
| ASV_436 | -8.015380866 | 4.23E-05 | Vicinamibacteraceae |
| ASV_261 | -8.061971646 | 4.42E-05 | Comamonadaceae |
| ASV_56 | -9.934757344 | 4.54E-05 | *Extensimonas* |
| ASV_705 | -7.198983447 | 5.05E-05 | Gammaproteobacteria |
| ASV_286 | -7.97622429 | 5.05E-05 | NS11-12 marine group |
| ASV_154 | -7.897518104 | 5.05E-05 | *Sediminibacterium* |
| ASV_419 | -7.91117207 | 6.42E-05 | Geobacteraceae |
| ASV_262 | -8.609091858 | 7.25E-05 | Sporichthyaceae |
| ASV_331 | -7.750439839 | 0.0001041 | *Lacibacter cauensis* |
| ASV_36 | 6.444765133 | 0.00011482 | *Streptomyces albidoflavus* |
| ASV_726 | -6.785907255 | 0.00011603 | Gammaproteobacteria |
| ASV_214 | -7.519134101 | 0.00011607 | Sporichthyaceae |
| ASV_437 | 7.416519245 | 0.00013279 | Rhodobacteraceae |
| ASV_128 | -8.509068444 | 0.00013553 | *Rubrivivax* |
| ASV_333 | -7.509117047 | 0.00014425 | NS11-12 marine group |
| ASV_164 | 7.142633493 | 0.00015092 | *Ralstonia pickettii* |
| ASV_231 | -8.205829225 | 0.00016897 | *Leptothrix* |
| ASV_442 | -7.342610314 | 0.00017265 | Cyanobacteriia |
| ASV_392 | -8.177801396 | 0.0002103 | *Terrimonas* |
| ASV_281 | -8.915080598 | 0.00024688 | *Paucibacter* |
| ASV_174 | -7.888482725 | 0.00025857 | *Prosthecobacter* |
| ASV_414 | -7.076134531 | 0.00027158 | Chloroflexi |
| ASV_269 | -8.002959627 | 0.00027158 | NS11-12 marine group |
| ASV_574 | -7.961154634 | 0.00028409 | NS11-12 marine group |
| ASV_491 | -6.431073132 | 0.00029637 | *Ahniella affigens* |
| ASV_268 | -7.874730247 | 0.00031784 | Comamonadaceae |
| ASV_531 | -5.89354426 | 0.00034495 | *Luteolibacter* |
| ASV_167 | 6.633958984 | 0.00038185 | *Sphingomonas leidyi* |
| ASV_87 | -7.651705802 | 0.00040691 | Candidatus Planktophila |
| ASV_90 | -8.446963814 | 0.00043338 | *Dinghuibacter* |
| ASV_143 | -7.600324309 | 0.00044168 | *Flavobacterium* |
| ASV_127 | -6.932066497 | 0.00046779 | Cyanobacteriia |
| ASV_215 | -6.735868543 | 0.00059451 | *Arenimonas* |
| ASV_525 | -7.518740568 | 0.00060633 | Sphingobacteriales |
| ASV_165 | -9.215645434 | 0.00061253 | Methylophilaceae |
| ASV_34 | -8.208225595 | 0.00069205 | Cyanobacteriia |
| ASV_276 | -7.192304144 | 0.00079722 | GKS98 freshwater group |
| ASV_560 | -7.322509219 | 0.00081525 | *Terrimonas* |
| ASV_997 | -6.659940447 | 0.00081776 | Gammaproteobacteria |
| ASV_401 | -7.279233624 | 0.0008179 | Beijerinckiaceae |
| ASV_117 | -8.893655887 | 0.00093391 | Cyanobacteriia |
| ASV_824 | -6.40202063 | 0.00093917 | Paludibaculum |
| ASV_4 | 6.111786487 | 0.00093917 | *Aeromonas salmonicida* |
| ASV_163 | -7.942648737 | 0.00093917 | *Tabrizicola* |
| ASV_598 | -6.527857494 | 0.00093917 | Bdellovibrionaceae |
| ASV_132 | -8.068896411 | 0.00093917 | Candidatus Aquirestis |
| ASV_206 | -7.172553633 | 0.00094247 | Candidatus Limnoluna |
| ASV_554 | -7.160433642 | 0.00102521 | Pedosphaeraceae |
| ASV_375 | -7.115646311 | 0.00103904 | *Emticicia* |
| ASV_762 | -7.882612942 | 0.00115557 | *Fluviicola* |
| ASV_351 | -7.013579776 | 0.0011633 | *Caulobacter* |
| ASV_183 | -6.281796294 | 0.00133877 | Verrucomicrobiae |
| ASV_607 | -7.703879155 | 0.00136722 | Candidatus Limnoluna |
| ASV_120 | -7.664763096 | 0.00138061 | *Polynucleobacter* |
| ASV_99 | -6.849266196 | 0.00140947 | *Brevundimonas* |
| ASV_29 | -5.992860552 | 0.00142877 | *Cyanobium PCC-6307* |
| ASV_655 | -7.558196415 | 0.00164124 | Gemmatimonadaceae |
| ASV_122 | -5.467603884 | 0.00170267 | Steroidobacteraceae |
| ASV_250 | 7.54044648 | 0.00176013 | *Clostridium sensu stricto 1 beijerinckii* |
| ASV_370 | -7.491989698 | 0.00186502 | Sphingobacteriales |
| ASV_344 | -6.046940943 | 0.00187358 | NS9 marine group |
| ASV_410 | -7.376342016 | 0.00195789 | Comamonadaceae |
| ASV_335 | -8.170864204 | 0.00206459 | *Methylotenera* |
| ASV_126 | -7.398975386 | 0.00210622 | *Flavobacterium* |
| ASV_834 | -7.300609908 | 0.00233556 | Microscillaceae |
| ASV_41 | -4.15859945 | 0.00238279 | *Arenimonas* |
| ASV_196 | -7.20169668 | 0.00245766 | *Armatimonas* |
| ASV_582 | -7.22266104 | 0.00249744 | Chitinophagaceae |
| ASV_82 | -4.626137436 | 0.00255397 | Steroidobacteraceae |
| ASV_385 | -6.448096419 | 0.00289094 | *Inhella* |
| ASV_566 | -7.006034731 | 0.00304998 | *Hirschia* |
| ASV_600 | -7.848461887 | 0.00311332 | *Cyanobium PCC-6307* |
| ASV_81 | -5.810095838 | 0.00311332 | Cyanobacteriia |
| ASV_941 | -7.011269698 | 0.00330816 | Latescibacterota |
| ASV_421 | -5.485526473 | 0.00340712 | *Blastocatella* |
| ASV_801 | -7.049433612 | 0.00340712 | Acidobacteriota |
| ASV_497 | -6.304551486 | 0.00340712 | Sphingobacteriales |
| ASV_197 | -7.675653876 | 0.00351007 | Gemmatimonadaceae |
| ASV_316 | -5.626439439 | 0.00361095 | *Dinghuibacter* |
| ASV_645 | -6.825995796 | 0.00377009 | *CL500-29 marine group* |
| ASV_229 | -7.577742771 | 0.00377009 | Candidatus Limnoluna |
| ASV_400 | -7.589799058 | 0.00383795 | *Dinghuibacter* |
| ASV_109 | -7.488966752 | 0.00418909 | *Flavobacterium sasangense* |
| ASV_509 | -7.479623587 | 0.00445165 | Sporichthyaceae |
| ASV_209 | -6.766230995 | 0.00445165 | Rickettsiales |
| ASV_147 | -7.367948771 | 0.00449055 | *Rhodobacter* |
| ASV_670 | -7.201807192 | 0.00594479 | Candidatus Planktoluna |
| ASV_52 | -7.201321981 | 0.00606616 | Sporichthyaceae |
| ASV_742 | -6.515898339 | 0.00646395 | Pedosphaeraceae |
| ASV_592 | 7.90202953 | 0.00651877 | Rhodobacteraceae |
| ASV_273 | -7.139405353 | 0.00654298 | CL500-29 marine group |
| ASV_288 | -5.473485532 | 0.00664844 | *Dechloromonas* |
| ASV_958 | 5.387560799 | 0.00688131 | *Pirellula* |
| ASV_800 | -7.77346728 | 0.00741052 | *Sphaerotilus* |
| ASV_352 | -6.380499074 | 0.00741052 | *Deefgea* |
| ASV_186 | -6.890174493 | 0.0081177 | Candidatus Planktoluna |
| ASV_372 | -6.931416443 | 0.00822464 | *Acidibacter* |
| ASV_105 | -7.565829998 | 0.0087315 | *Rhodoferax* |
| ASV_750 | -6.417608197 | 0.00895658 | *Fluviicola* |
| ASV_666 | -6.816303561 | 0.00899545 | *Ferruginibacter* |
| ASV_264 | -6.795236563 | 0.00936493 | *Algoriphagus* |
| ASV_657 | -6.774326455 | 0.00952247 | Bacteroidetes vadinHA17 |
| ASV_904 | -6.716579533 | 0.01082709 | *Flavobacterium* |
| ASV_292 | -6.649900802 | 0.01107234 | Candidatus Methylopumilus |
| ASV_340 | -7.324881037 | 0.01109203 | *Hydrogenophaga* |
| ASV_756 | -7.311457636 | 0.0115705 | Methylophilaceae |

Table S12: Differential ASVs identified between Red shiner and Water samples

| **ASV_ID** | **log2FoldChange** | **padj** | **Lowest classification** |
| --- | --- | --- | --- |
| ASV_17 | 28.38416436 | 1.02E-25 | Cyanobacteriia |
| ASV_104 | 25.02000726 | 3.25E-19 | Bacilli |
| ASV_25 | -26.64983866 | 1.44E-17 | Cyanobacteriia |
| ASV_180 | -25.44307973 | 1.66E-16 | Comamonadaceae |
| ASV_342 | -25.05575759 | 4.07E-16 | Cyanobacteriia |
| ASV_1 | 7.618708456 | 1.01E-15 | *Roseomonas gilardii* |
| ASV_297 | 23.233665 | 4.67E-15 | *Pseudomonas fragi* |
| ASV_43 | -26.54758525 | 4.93E-15 | Cyanobacteriia |
| ASV_7 | -26.7138153 | 4.93E-15 | Cyanobacteriia |
| ASV_141 | -24.67287299 | 1.55E-13 | *Cyanobium PCC-6307* |
| ASV_407 | -24.54894437 | 2.10E-13 | Sporichthyaceae |
| ASV_396 | -24.36256184 | 2.69E-13 | Comamonadaceae |
| ASV_363 | -24.23700379 | 3.54E-13 | *Hydrogenophaga* |
| ASV_638 | -24.105766 | 4.00E-13 | *Polynucleobacter cosmopolitanus* |
| ASV_5 | -11.70280327 | 6.48E-12 | Sporichthyaceae |
| ASV_12 | -11.33203205 | 5.11E-11 | Sporichthyaceae |
| ASV_36 | 8.695732494 | 7.92E-11 | *Streptomyces albidoflavus* |
| ASV_623 | 22.36909604 | 1.90E-10 | Coriobacteriales |
| ASV_747 | 22.24543694 | 2.26E-10 | Candidatus Saccharimonas |
| ASV_3 | -10.85061905 | 2.33E-10 | *Polynucleobacter asymbioticus* |
| ASV_20 | -10.59395254 | 5.25E-10 | *Limnohabitans* |
| ASV_19 | -10.29488393 | 6.02E-10 | *Pseudarcicella* |
| ASV_2 | -11.29615238 | 1.34E-09 | Comamonadaceae |
| ASV_9 | -10.43916387 | 1.40E-09 | Comamonadaceae |
| ASV_18 | -10.35274481 | 4.84E-09 | Cyanobacteriia |
| ASV_31 | -9.91961843 | 8.04E-09 | Candidatus Planktophila |
| ASV_32 | -9.88874757 | 1.33E-08 | *Polynucleobacter cosmopolitanus* |
| ASV_46 | -9.966698285 | 1.39E-08 | Sediminibacterium |
| ASV_65 | -9.699743632 | 3.33E-08 | *Flavobacterium cheonhonense* |
| ASV_58 | -9.320581714 | 8.04E-08 | Candidatus Methylopumilus |
| ASV_60 | -9.449983871 | 8.04E-08 | Cyanobacteriia |
| ASV_93 | -9.072729781 | 2.68E-07 | *Limnohabitans* |
| ASV_167 | 8.495656567 | 3.75E-07 | *Sphingomonas leidyi* |
| ASV_71 | -8.83006081 | 4.29E-07 | *Methylotenera* |
| ASV_73 | -8.738623404 | 5.85E-07 | *Novosphingobium* |
| ASV_72 | -8.690650418 | 8.10E-07 | Microbacteriaceae |
| ASV_176 | -8.30264416 | 2.39E-06 | Microbacteriaceae |
| ASV_42 | -9.274122434 | 2.69E-06 | Comamonadaceae |
| ASV_113 | -9.69057658 | 2.89E-06 | *Pseudanabaena PCC-7429* |
| ASV_164 | 8.670604127 | 2.92E-06 | *Ralstonia pickettii* |
| ASV_44 | -10.36943861 | 3.34E-06 | *Ramlibacter* |
| ASV_103 | -9.026412858 | 5.03E-06 | *Rhodoluna* |
| ASV_11 | -8.991672109 | 5.94E-06 | *Acidovorax facilis* |
| ASV_122 | -7.959473045 | 6.40E-06 | Steroidobacteraceae |
| ASV_70 | -9.826829697 | 1.03E-05 | Cyanobacteriia |
| ASV_40 | -8.579774136 | 1.50E-05 | Sporichthyaceae |
| ASV_85 | -9.657316958 | 1.72E-05 | Sporichthyaceae |
| ASV_53 | -8.536863857 | 1.72E-05 | *Sphingorhabdus* |
| ASV_282 | -7.586665318 | 2.30E-05 | Blfdi19 |
| ASV_54 | -8.365196979 | 2.61E-05 | *Polynucleobacter* |
| ASV_116 | -8.215774807 | 3.81E-05 | *Rhodoluna* |
| ASV_188 | -8.171584279 | 3.86E-05 | *Methylotenera* |
| ASV_94 | -9.148866335 | 3.97E-05 | Comamonadaceae |
| ASV_112 | -8.002757759 | 5.50E-05 | Candidatus Aquiluna |
| ASV_107 | -7.305706924 | 5.61E-05 | *CL500-29 marine group* |
| ASV_158 | -7.072700726 | 7.40E-05 | *Prosthecobacter* |
| ASV_96 | -8.877021322 | 8.72E-05 | *Fluviicola* |
| ASV_125 | -6.95055291 | 1.00E-04 | Candidatus Planktoluna |
| ASV_220 | -7.873586381 | 0.00010278 | Cyanobacteriia |
| ASV_173 | -7.707541578 | 0.00011297 | *Sphingorhabdus* |
| ASV_102 | -8.651074096 | 0.00011534 | Rhodocyclaceae |
| ASV_47 | -8.512154601 | 0.00013731 | *Flavobacterium* |
| ASV_41 | -7.421457559 | 0.00013811 | *Arenimonas* |
| ASV_193 | -7.630719934 | 0.0001402 | *Dinghuibacter* |
| ASV_467 | 7.060088623 | 0.00016507 | *Dietzia lutea* |
| ASV_22 | -8.387920192 | 0.0001798 | *Flavobacterium saccharophilum* |
| ASV_178 | -7.38657299 | 0.00020236 | Rhodobacteraceae |
| ASV_387 | -6.741190717 | 0.00021599 | Methylomonadaceae |
| ASV_360 | -6.586620495 | 0.00023628 | Vicinamibacteraceae |
| ASV_156 | -7.433543739 | 0.00027759 | *Rhizorhapis* |
| ASV_377 | -7.235087008 | 0.00028786 | *Altererythrobacter* |
| ASV_288 | -7.92221726 | 0.00034848 | *Dechloromonas* |
| ASV_119 | -8.719020596 | 0.00041767 | Comamonadaceae |
| ASV_373 | 6.909674165 | 0.00047208 | *Brevibacterium aurantiacum* |
| ASV_239 | -8.61996221 | 0.00059342 | Chitinophagaceae |
| ASV_312 | -7.559369541 | 0.00063303 | Cyanobacteriia |
| ASV_263 | -7.485636678 | 0.00073238 | *Novosphingobium* |
| ASV_82 | -6.006327403 | 0.0007353 | Steroidobacteraceae |
| ASV_74 | -6.788265768 | 0.0007647 | Candidatus Aquirestis |
| ASV_218 | -7.406611683 | 0.00086774 | *Sediminibacterium* |
| ASV_242 | -7.469382253 | 0.00086816 | *Dinghuibacter* |
| ASV_378 | -6.667463203 | 0.00091356 | Microscillaceae |
| ASV_327 | -7.363567637 | 0.00091519 | *Methylotenera* |
| ASV_152 | -7.214424768 | 0.00113511 | Candidatus Planktophila |
| ASV_436 | -7.224139688 | 0.00116694 | Vicinamibacteraceae |
| ASV_286 | -6.880354743 | 0.00119099 | NS11-12 marine group |
| ASV_253 | -6.52742092 | 0.00120929 | *Lacihabitans* |
| ASV_262 | -7.835277473 | 0.00136525 | Sporichthyaceae |
| ASV_154 | -7.048977985 | 0.00147801 | *Sediminibacterium* |
| ASV_421 | -6.399812704 | 0.00170408 | *Blastocatella* |
| ASV_174 | -7.766585128 | 0.00177693 | *Prosthecobacter* |
| ASV_419 | -6.965806834 | 0.00182406 | Geobacteraceae |
| ASV_705 | -6.276496134 | 0.00200476 | Gammaproteobacteria |
| ASV_128 | -7.607654022 | 0.00223556 | *Rubrivivax* |
| ASV_172 | -6.006805126 | 0.00223556 | Sutterellaceae |
| ASV_331 | -6.883435967 | 0.00223556 | *Lacibacter cauensis* |
| ASV_333 | -6.817464815 | 0.00245423 | NS11-12 marine group |
| ASV_90 | -8.25721799 | 0.00245423 | *Dinghuibacter* |
| ASV_214 | -6.678187859 | 0.00266722 | Sporichthyaceae |
| ASV_231 | -7.360889198 | 0.00271534 | *Leptothrix* |
| ASV_64 | -6.596460707 | 0.00279387 | *Acidovorax carolinensis* |
| ASV_281 | -7.998423483 | 0.00313615 | *Paucibacter* |
| ASV_442 | -6.587233775 | 0.00319525 | Cyanobacteriia |
| ASV_215 | -6.598246403 | 0.00324691 | Arenimonas |
| ASV_726 | -5.943006909 | 0.00336654 | Gammaproteobacteria |
| ASV_531 | -5.222955661 | 0.00384264 | *Luteolibacter* |
| ASV_185 | 7.781111737 | 0.00421211 | *Leucobacter* |
| ASV_269 | -7.046499931 | 0.00426776 | NS11-12 marine group |
| ASV_414 | -6.312291025 | 0.00469807 | Chloroflexi |
| ASV_184 | -6.156636826 | 0.00513132 | Gammaproteobacteria |
| ASV_268 | -6.88860155 | 0.00513132 | Comamonadaceae |
| ASV_87 | -6.843338355 | 0.00530024 | Candidatus Planktophila |
| ASV_276 | -6.860388354 | 0.00545031 | *GKS98 freshwater group* |
| ASV_165 | -8.26360797 | 0.00557867 | Methylophilaceae |
| ASV_392 | -6.534738756 | 0.00579815 | Terrimonas |
| ASV_143 | -6.731189641 | 0.00598371 | Flavobacterium |
| ASV_491 | -5.575633854 | 0.00656396 | Ahniella affigens |
| ASV_34 | -7.361191089 | 0.00656396 | Cyanobacteriia |
| ASV_574 | -6.375355027 | 0.00705528 | NS11-12 marine group |
| ASV_117 | -8.038640619 | 0.00705737 | Cyanobacteriia |
| ASV_127 | -6.023924461 | 0.00753938 | Cyanobacteriia |
| ASV_525 | -6.592848735 | 0.00753938 | Sphingobacteriales |
| ASV_163 | -7.113491904 | 0.00853983 | *Tabrizicola* |
| ASV_132 | -7.164733315 | 0.00866224 | Candidatus Aquirestis |
| ASV_401 | -6.42536816 | 0.00918217 | Beijerinckiaceae |
| ASV_560 | -6.373103659 | 0.00989243 | *Terrimonas* |
| ASV_206 | -6.365026973 | 0.00997187 | Candidatus Limnoluna |

Table S13: Differential ASVs identified between Sand shiner and Red Shiner

| **ASV_ID** | **log2FoldChange** | **padj** | **Lowest classification** |
| --- | --- | --- | --- |
| ASV_138 | -24.56769416 | 5.37E-17 | Cyanobacteriia |
| ASV_318 | -25.07408153 | 9.76E-14 | *Lactobacillus* |
| ASV_169 | 22.57507991 | 2.91E-11 | *Enterococcus* |
