# Supplementary figures and images for: Gut Microbial Composition of Cyprinella lutrensis (Red Shiner) and Notropis stramineus (Sand Shiner): Insights from Wild Fish Populations
Source: Microb Ecol. 2024 May 22;87(1):75. doi: 10.1007/s00248-024-02386-z (PMC11111511; doi:10.1007/s00248-024-02386-z)

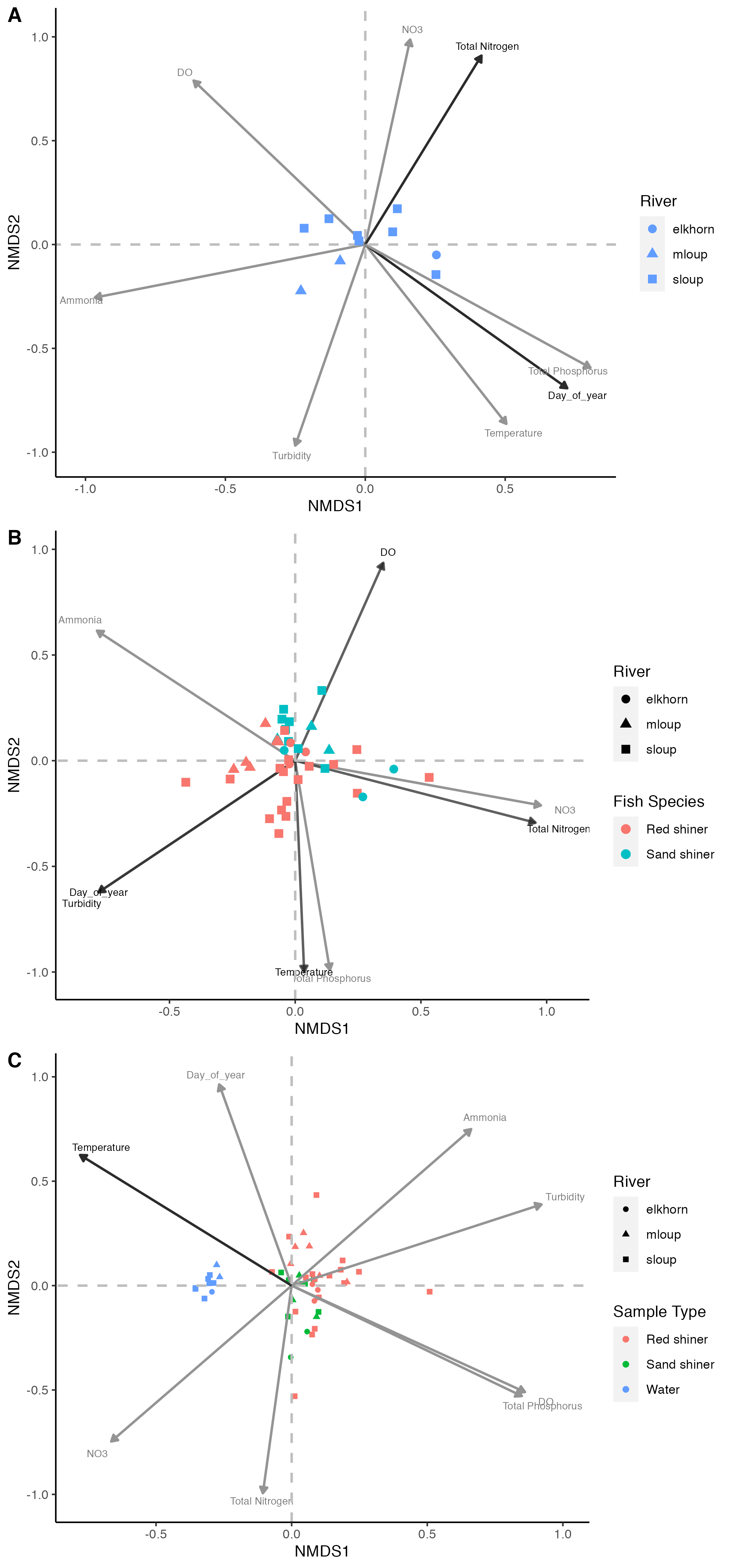

Supplement: Supplementary file 1 — Supplementary file1 (PNG 469 KB) [file 248_2024_2386_MOESM1_ESM.png]
